# Use of malaria rapid diagnostic tests by community health workers in Afghanistan: cluster randomised trial

**DOI:** 10.1186/s12916-017-0891-8

**Published:** 2017-07-07

**Authors:** Toby Leslie, Mark Rowland, Amy Mikhail, Bonnie Cundill, Barbara Willey, Asif Alokozai, Ismail Mayan, Anwar Hasanzai, Sayed Habibullah Baktash, Nader Mohammed, Molly Wood, Habib-u-Rahman Rahimi, Baptiste Laurent, Cyril Buhler, Christopher J. M. Whitty

**Affiliations:** 1 London School of Hygiene & Tropical Medicine, London, WC1H 7HT UK; 2 Health Protection and Research Organisation, Kabul, Afghanistan; 3 HealthNet-TPO, Kabul, Afghanistan; 4 Merlin, Kunduz, Afghanistan; 5 OR Diagnostics, Paris, France

**Keywords:** Malaria, Rapid diagnostic test, Afghanistan, Community health worker, Cluster randomised trial

## Abstract

**Background:**

The World Health Organisation (WHO) recommends parasitological diagnosis of malaria before treatment, but use of malaria rapid diagnostic tests (mRDTs) by community health workers (CHWs) has not been fully tested within health services in south and central Asia. mRDTs could allow CHWs to diagnose malaria accurately, improving treatment of febrile illness.

**Methods:**

A cluster randomised trial in community health services was undertaken in Afghanistan. The primary outcome was the proportion of suspected malaria cases correctly treated for polymerase chain reaction (PCR)-confirmed malaria and PCR negative cases receiving no antimalarial drugs measured at the level of the patient. CHWs from 22 clusters (clinics) received standard training on clinical diagnosis and treatment of malaria; 11 clusters randomised to the intervention arm received additional training and were provided with mRDTs. CHWs enrolled cases of suspected malaria, and the mRDT results and treatments were compared to blind-read PCR diagnosis.

**Results:**

In total, 256 CHWs enrolled 2400 patients with 2154 (89.8%) evaluated. In the intervention arm, 75.3% (828/1099) were treated appropriately vs. 17.5% (185/1055) in the control arm (cluster adjusted risk ratio: 3.72, 95% confidence interval 2.40–5.77; *p* < 0.001). In the control arm, 85.9% (164/191) with confirmed *Plasmodium vivax* received chloroquine compared to 45.1% (70/155) in the intervention arm (*p* < 0.001). Overuse of chloroquine in the control arm resulted in 87.6% (813/928) of those with no malaria (PCR negative) being treated vs. 10.0% (95/947) in the intervention arm, *p* < 0.001. In the intervention arm, 71.4% (30/42) of patients with *P. falciparum* did not receive artemisinin-based combination therapy, partly because operational sensitivity of the RDTs was low (53.2%, 38.1–67.9). There was high concordance between recorded RDT result and CHW prescription decisions: 826/950 (87.0%) with a negative test were not prescribed an antimalarial. Co-trimoxazole was prescribed to 62.7% of malaria negative patients in the intervention arm and 15.0% in the control arm.

**Conclusions:**

While introducing mRDT reduced overuse of antimalarials, this action came with risks that need to be considered before use at scale: an appreciable proportion of malaria cases will be missed by those using current mRDTs. Higher sensitivity tests could be used to detect all cases. Overtreatment with antimalarial drugs in the control arm was replaced with increased antibiotic prescription in the intervention arm, resulting in a probable overuse of antibiotics.

**Trial registration:**

ClinicalTrials.gov, NCT01403350. Prospectively registered.

**Electronic supplementary material:**

The online version of this article (doi:10.1186/s12916-017-0891-8) contains supplementary material, which is available to authorized users.

## Background

Acute febrile illness is one of the commonest presentations to clinics in Asia, with malaria being one of multiple possible causes. Malaria rapid diagnostic tests (mRDTs) are increasingly used across health service settings to improve diagnosis and treatment of febrile illness by detecting malaria [1]. The World Health Organisation (WHO) recommends universal coverage with diagnostic testing for malaria to ensure that patients are appropriately prescribed artemisinin combination therapy (ACT) and other antimalarial drugs [2] and improving treatment of other causes of febrile illness. Wider use of mRDTs should also improve disease surveillance, particularly in areas of low to moderate transmission where malaria elimination is being considered.

There is now reasonable evidence to support WHO policy on the effect of using falciparum-specific RDTs in highly endemic areas in Africa, where almost all malaria is caused by *Plasmodium falciparum* [3–7]. As a result, RDTs are now widely used in Africa, and their use is growing [8]. There is much less evidence from Asia, where more than one billion people live in malaria endemic areas [9] which are co-endemic for *P. falciparum* and *P. vivax* and where the proportion of febrile patients who have malaria is generally much lower than in Africa. Afghanistan is typical of much of low-resource south Asia, where *P. vivax* makes up around 85–95% of malaria cases [10].

Community health workers (CHWs) are often the first point of contact with healthcare services for those with fever or a history of fever. In common with much of south Asia, CHWs in Afghanistan have limited training and do not have access to laboratory testing. Currently malaria reported in this setting is diagnosed using clinical signs and symptoms alone which are indistinguishable from many other causes of fever. This is termed ’suspected malaria’ in training curricula and treatment guidelines and is often treated presumptively with chloroquine (CQ) and/or sulphadoxine-pyrimethamine (SP) since there is no way of distinguishing the species of malaria necessary to guide treatment. This approach leads to misdiagnosis and overtreatment of malaria, sometimes resulting in 100% mistreatment and antimalarial wastage in areas of very low malaria endemicity [11, 12]. In initial testing in health facilities where healthcare workers have formal training, an earlier study in this Afghan setting showed that diagnosis using mRDTs led to improved prescribing of antimalarial drugs at an individual level and an increase in prescribing of antibiotics [11].

We therefore conducted a two-arm, stratified cluster randomised trial amongst communities within the formal health service in two provinces of Afghanistan to investigate the effect of training and the use of mRDTs on accurate diagnosis and appropriate prescribing of antimalarials and antibiotics by CHWs compared to normal clinical diagnosis. A cluster randomised design was chosen because a patient randomised trial in the setting of CHWs is unlikely to give accurate estimates of effect. This happens because prescribers influence one another (a healthcare worker community effect), and perceptions of interventions may influence normal practice by the community (a patient community effect) [13].

## Methods

### Study setting

Clinics were purposively selected if the location was relatively secure, part of the formal public health system and provided outpatient care for febrile illnesses, including malaria [11]. Twenty-two clinics (clusters) were selected. Twelve of these were in an area defined as being of ’moderate transmission’ in the National Malaria Strategy [10] in the eastern region of Afghanistan (malaria incidence of 1–10 per 1000 per year). Ten clinics were in an area defined as being of low transmission in northern Afghanistan (<1 per 1000 per year) [10–12].

The Afghan public health system comprises Basic Health Centres (BHC), Comprehensive Health Centres (CHC) and District Hospitals. Our study clinics were all BHCs or CHCs which provide outpatient services for acute illnesses. Each health centre employs CHWs to serve a population of 5000–10,000 people each. CHWs perform a range of curative and preventative tasks including treatment of acute illnesses, antenatal care, vaccination (under the Extended Programme of Immunisation) and health education and awareness. They are often the first point of contact with health services, especially in rural and poorer areas. CHWs are volunteers who do not receive a wage (although they sometimes receive payments for services, such as completing national surveys). Being a CHW is not a full-time task, and CHWs are often teachers, farmers or religious leaders or have other occupations. They receive a 6-week basic training before being deployed, but also receive additional training provided by the government or non-governmental organisations (NGOs) in various guidelines and approaches to community health work. The approaches they follow are based on Integrated Management of Childhood Illness (IMCI) guidelines and definitions, although it is unknown how well these guidelines are applied in practice. CHWs are supervised by a community health manager who supplies basic items, including essential medicines. CHWs keep records of consultations and activities which are entered into the national health information system. They operate, normally, as a married couple from a health post or house. BHCs and CHCs were used as the units of randomisation (cluster) in this study.

### Sample size and randomisation

Introducing RDTs to community centres with associated training is a major public health effort, so it would only be justified with a substantial public health benefit. Based on randomisation of 22 clusters, 2542 individuals would be required to detect an absolute increase in the primary outcome (appropriate treatment) from 10% in the control arm (from previous observational data) to 50% in the intervention arm with 80% power at the 5% significance level, assuming a between-cluster coefficient of variation (κ) of 0.5. The sample size was weighted according to the total number of consultations per cluster to give an overall harmonic mean of 68 patients per cluster.

Clinics were stratified by province and randomised within strata through a process of restricted (constrained) randomisation, to ensure marginal balance across strata and study arms on covariates expected to be important correlates of the primary outcome [14]. Balance was achieved for the following: total sample size per arm differing by no more than 50; harmonic mean number of patients per cluster between 65 and 71; number of health posts (CHW houses) per arm differing by no more than 10. These restriction criteria led to 1589 acceptable allocations, of which one was chosen at random. Randomisation was conducted by one of the trial statisticians (BC), who was not involved in the delivery of the intervention or assessment or final analysis of the study outcomes, using a program written in R statistical software version 2.13.0.

### Intervention and control arms

The two arms of the trial were (1) training on the national guidelines for community-based management of malaria (control) and (2) the same training as the control plus additional training in the use of RDTs and provision of RDTs for use in practice (intervention). A description of the control and intervention arms is given in Table 1.

**Table 1:** Intervention description

Training/intervention	description	Control arm (no RDTs)	Intervention arm (RDTs)
Basic CHW training	6-week basic training in preventative and curative services for infectious diseases, nutrition and mother-and-child health. Malaria and malaria treatment guidelines are included in the curriculum. Includes record keeping for health service reporting (Health Management Information System, HMIS)	√	√
Malaria training	1-day refresher workshop on control, treatment and surveillance for malaria, including the guidelines for community-based treatment of suspected malaria	√	√
mRDT training	Half-day training on use of mRDTs, including symptoms, practical and demonstrated use of mRDTs and treatment guidelines for RDT-confirmed *P. falciparum*	X	√
mRDTs	Bivalent immunofluorescent antigen detection tests for diagnosis of *P. falciparum* (histidine-rich protein 2, HRP2) and pan-specific Plasmodium lactate dehydrogenase (pLDH). See text for manufacturer details	X	√
ACTs	Sulphadoxine-pyrimethamine with artesunate according to standard dosing table for adults and children in co-blister packs. For treatment of RDT-confirmed *P. falciparum*	X	√
SP and CQ	For treatment of suspected malaria (SP and/or CQ) and treatment of pan-specific positive and *P. falciparum* negative RDTs	√	√
Co-trimoxazole	For treatment of pneumonia in children; included in the standard package of drugs provided to CHWs	√	√

The appropriate training for the study arm was conducted at the clinics prior to data collection by national trainers who were not part of the study team, and this training was designed to be possible for a malaria control programme to provide routinely. Some training sessions were observed by study staff (IM) to ensure that it was done according to the training curriculum. All CHWs associated with the study clinics were eligible for the training.

The 1-day training curriculum for all CHWs in both the control and intervention arms covered the basics of malaria and transmission, diagnosis of suspected malaria according to clinical signs and symptoms, national treatment guidelines and the national strategy to control malaria. In the control arm, CHWs had a stock of CQ and SP for treatment of suspected malaria. In the intervention arm, CHWs additionally received a practical module on the use of mRDTs and provision of ACT treatment (sulphadoxine/pyrimethamine and artesunate, SP/AS) for cases of mRDT positive *P. falciparum* and CQ for mRDT positive pan-specific (assumed to be *P. vivax* malaria). The module was based on WHO guidelines [2] and written by a National Malaria Programme Task Force. CHWs were given a take-home reference sheet with pictorial instructions on how to use the mRDT and provide treatment (Additional file 1). After the training, CHWs were provided with mRDTs, drugs and other materials for using mRDTs and applying treatment. The mRDTs were CareStart Malaria HRP2/pLDH (Pf/pan) Combo Tests, approved by WHO-FIND [15, 16]. Supplies were replenished when needed.

### Data collection and participant enrolment

Two weeks after training, all CHWs from both study arms were recalled and trained in study data collection methods if they gave consent to participate in the study. They were instructed on use of a pre-tested semi-pictorial form for recording data on each patient consultation (Additional file 1). This included details of age and gender, symptoms and their time of onset, diagnosis, treatment given and referral. The intervention arm form had space for recording mRDT results (Additional file 1). Training was given on blood safety, including information on standard precautions and safe disposal of sharps and blood-contaminated materials.

Patients of any age were enrolled by CHWs if they gave informed consent and presented with symptoms suspected to be due to malaria (fever or history of fever and chills). Patients were excluded if they had sought care for the episode of illness from any other source; if the CHW referred the patient directly to the clinic for any reason prior to diagnosis; or if they had any signs of severe illness. Patients who were not enrolled received care according to standard guidelines. Patients exited the trial at the end of the consultation with the CHW. No further formal follow-up was required by the protocol due to the operational difficulties of doing so in Afghanistan.

### Laboratory procedures

All patients had a blood-spot filter paper (Whatman 3MM chromatography paper) collected at enrolment by the CHW; this was used to provide the gold standard polymerase chain reaction (PCR) diagnosis used to define the outcomes. CHWs in Afghanistan do not take and store malaria blood slides, so it was not possible to collect them from patients in the trial. Filter-paper blood samples were analysed using standard PCR methods outlined previously [11]. The PCR analysis was batched and undertaken blind to study arm allocation at the National Malaria Control Programme Laboratories in Kabul.

### Outcome measures

The primary outcome was the proportion of patients appropriately treated with antimalarial drugs by the CHWs. This was a composite measure defined against the malaria PCR result for each patient: falciparum or mixed-species malaria treated with SP/AS, vivax malaria treated with CQ (regardless of accompaniment with SP) and PCR-confirmed negative cases receiving no antimalarial drug.

Secondary outcomes were as follows: antimalarial treatment accuracy disaggregated by species (i.e. proportion of falciparum cases receiving ACT, proportion of vivax malaria cases receiving CQ); proportion of malaria (PCR) negative cases receiving antimalarials and the proportion receiving antibiotics; and prescription of antimalarials by recorded RDT result. An additional analysis was undertaken in those under 5 and those over 5 years of age. The study also evaluated the accuracy of the mRDT against PCR results and the concordance between the mRDT result given by the CHW and a second read by the trained study registrar, during which we used a WHO-FIND and nationally approved bivalent test [15, 16] capable of detecting pan-specific Plasmodium lactase dehydrogenase (pLDH) and *P. falciparum*-specific histidine-rich protein 2 (HRP2).

### Statistical analysis

Data were double entered into an MS Access database and analysed using STATA v12 (2011, StataCorp., College Station, TX, USA). The analysis was conducted on an intention-to-treat basis, and the effect of the intervention was analysed using methods suitable for cluster randomised trials with fewer than 20 clusters per arm [17]. The observed percentage treated appropriately was calculated for each clinic (cluster), and the mean of the cluster-level estimates was taken to give the prevalence of the primary outcome in each arm. Due to the skewness in the distribution of the cluster proportions, a log transformation was applied, and the mean of the log proportions was estimated in each arm and stratum. Given the equal number of clusters allocated to the study arms within each stratum, the risk ratio (RR) for the intervention effect was computed from exponentiation of the difference between the mean of the log proportions in each arm. Corresponding 95% confidence intervals (CIs) for the RRs were based on the logarithm of the RR, and a stratified *t* test was used to test the null hypothesis of no overall intervention effect. The pooled variance of the log proportions within each stratum-arm combination was estimated as the residual mean square from a two-way analysis of variance (ANOVA) incorporating the stratum by study arm interaction. An estimate of the between-cluster variation, *k*, was calculated [17].

The main analyses were adjusted for pre-defined potential confounders, based on information from characteristics of clusters, CHWs and patients and the way in which they differed by trial arm at baseline. A range of confounding factors were considered for inclusions in the model, but were not independently associated with the main outcome and so were not included in the final analysis. These included patient-level factors (age, gender, pregnancy, symptoms at presentation, days since onset of symptoms); CHW-level factors (gender, age, socioeconomic group of the CHW, length of service, training history) and cluster-level factors (clinic type (CHC/BHC); malaria slide positivity rate).

Adjustment for covariates was made by fitting a logistic regression model using data on individuals, and including the covariates of interest and the intervention effect. Expected numbers with appropriate treatment were computed, in the absence of the intervention for intervention clinics, and compared with the observed values to provide ratio residuals for each clinic. Risk ratios, 95% CIs and hypothesis testing were calculated using the above methods, with the residuals replacing cluster-specific proportions.

### Ethics and trial registration

The trial protocol was reviewed and approved by the ethics committee of the Institutional Review Board, Ministry of Public Health, Afghanistan, the Ethics Committee of the London School of Hygiene & Tropical Medicine and a Data Safety Monitoring Board. The trial was prospectively registered at ClinicalTrials.gov, NCT01403350.

## Results

Patients were enrolled between October 2011 and May 2012, 22 clinics were randomised and of the 256 CHWs, 222 (86.7%) received training in the study data collection methods, consented and enrolled patients. Those who did not enrol patients were either not present for training or did not give consent to participate. The trial ended when the sample size had been reached.

### Enrolment characteristics

**Fig. 1:**
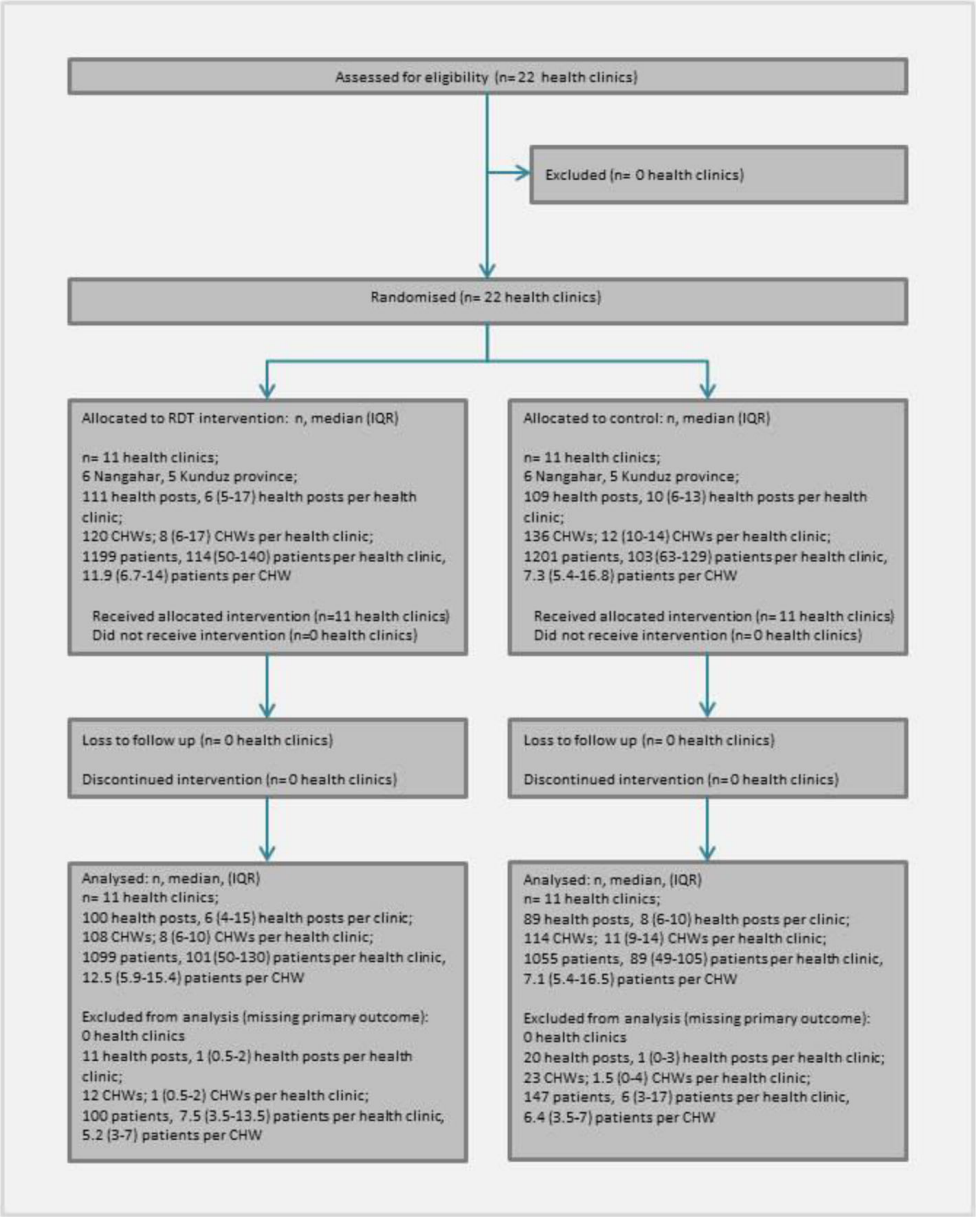
Trial profile

Table 2 shows the characteristics of the clinics, health posts, CHWs and patients enrolled in the study, and Fig. 1 shows the trial profile. Most key characteristics differed minimally between the control and intervention arms of the trial.

**Table 2:** Enrolment characteristics of clinics, CHWs and patients by trial arm

	Intervention	Control
Clinic-level characteristics:		
Number of clinics	11	11
Number of clinics per province		
Kunduz	5	5
Nangahar	6	6
Number of health posts (median number per clinic, interquartile range (IQR))	111 (6, 5–17)	109 (10, 6–13)
Number of patients enrolled (median number per clinic, IQR)	1199 (114, 50–140)	1201 (103, 63–129)
Health post and CHW-level characteristics:		
Number of CHWs (median number of CHWs per health clinic, IQR)	120 (8, 6–17)	137 (12, 10–14)
Included in analysis	108	114
CHW gender (*n*, % male)	75 (62.5)	83 (60.6)
Education level of CHWs, *n* (%)		
None	41 (34.8)	34 (25.0)
Informal education	5 (4.2)	5 (3.7)
Primary	20 (17.0)	20 (14.7)
Secondary	44 (37.3)	42 (30.9)
Post-secondary/higher	8 (6.8)	35 (25.7)
Socioeconomic status of CHWs (*n*, %)		
Below median	59 (51.3)	63 (48.1)
Above median	56 (48.7)	68 (51.9)
Data missing	5	5
Median number of consultations in week prior to the study (IQR)	11 (7–20)	14 (9–19)
Median number of hours in last week performing CHW activities (IQR)	2.5 (1.3–5.7)	5.4 (1.8–9.0)
Patient characteristics:		
Number of patients (median per CHW, IQR)	1199 (11.9, 6.7–14)	1201 (7.3, 5.4–16.8)
Number of patients evaluated:	1099	1055
Reasons for exclusion:		
Missing treatment or diagnosis data	81	128
Reference diagnosis missing	19	19
Prevalence of malaria in the sample (*n*, %)		
*P. vivax*	159 (13.2)	194 (16.2)
*P. falciparum*	45 (3.7)	45 (3.7)
Mixed infection	2 (0.2)	1 (0.1)
Gender of patient (*n*, % male)	647 (54.6)	674 (56.2)
Age band of patient (*n*, %)		
0–1 year	9 (0.8)	5 (0.4)
1–5 years	82 (6.9)	62 (4.2)
6–10 years	269 (22.8)	171 (14.3)
10–18 years	302 (25.6)	311 (26.0)
> 18 years	520 (44.0)	649 (54.2)
*N*, % of patients enrolled by male CHW	975 (81.5)	822 (68.6)
*N*, % of patients enrolled at time of day		
Morning (6 am–12 pm)	425 (35.5)	477 (39.8)
Afternoon (12 pm–6 pm)	338 (28.2)	319 (26.6)
Evening (6 pm–10 pm)	354 (29.5)	354 (29.5)
Night (11 pm–6 am)	82 (6.8)	50 (4.2)
*N*, % of patients enrolled by location		
Health post	959 (81.4)	803 (68.5)
Patient’s home	207 (17.6)	362 (30.4)
Other	12 (1.0)	25 (2.1)
Reported symptoms (*n*, %)		
Fever	1119 (93.5)	1172 (97.6)
Headache	1113 (92.8)	1119 (93.2)
Vomiting	311 (26.0)	488 (40.7)
Diarrhoea	169 (14.1)	252 (21.0)
Cough	569 (47.5)	416 (34.6)
Other symptom(s)	210 (17.5)	131 (10.9)
Patients with fast breathing (*n*, %)	57 (4.8)	26 (2.2)

Data from 246/2400 patients (10.1%) were not evaluable either because of missing reference diagnoses (*n* = 38) or missing outcome data (diagnosis and/or treatment not recorded on the case record form) (*n* = 209).

### Primary outcome

**Fig. 2:**
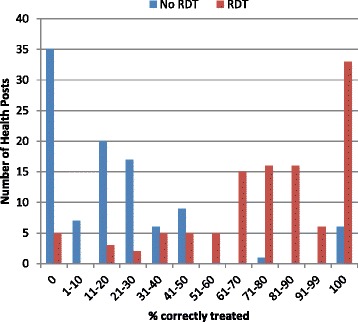
Effect of malaria RDTs on accuracy of malaria treatment at health post level

The proportion of patients appropriately treated for malaria was substantially higher in the intervention arm (828/1099, 75.3%) than in the control arm (185/1055, 17.5%); after adjusting for study design the risk ratio was 3.72 (95% CI: 2.40–5.77, *p* < 0.001) (Table 3). The proportion of health posts that provided accurate treatment to fewer than 50% of all patients who presented was 94/101 (93.1%) in the control arm and 20/111 (18.0%) in the intervention arm (Fig. 2).

**Table 3:** Primary and secondary outcomes by trial arm

Patient level: *n*, (%)	Intervention *n* = 1099	Control *N* = 1055	Risk ratio (95% CI)	*p* value
Primary outcome				
Appropriate treatment of malaria by CHWs^a^	828 (75.3)	185 (17.5)	3.72 (2.40–5.77)	<0.001
Secondary outcomes				
Malaria negative patients prescribed antimalarial drug (*n* = 1875)	95 (10.0)	813 (87.6)	0.11 (0.08–0.15)	<0.001
P. falciparum malaria cases prescribed an ACT (*n* = 86)	12 (28.6)	–^b^	–	
P. vivax malaria cases prescribed CQ (*n* = 346)	70 (45.1)	164 (85.9)	0.43 (0.31–0.59)	<0.001
Malaria negative patients prescribed co-trimoxazole (*n* = 1756)	613 (67.2)	295 (35.0)	1.98 (1.18–3.33)	0.012
Referral practices:				
Referral on to formal health services (*n* = 2399)	414 (34.6)	317 (26.4)	1.53 (0.89–2.63)	0.116

^a^Composite measure defined against the malaria PCR result for each patient: falciparum or mixed-species malaria treated with SP/AS; vivax malaria treated with CQ (regardless of accompaniment with SP); and PCR-confirmed negative cases receiving no antimalarial drug

^b^ACTs were not used in the control arm, as they can only be prescribed based on parasitological diagnosis. In the intervention arm, *N* = 42

### Secondary outcomes

#### Targeting of antimalarial drugs and use of the antibiotic co-trimoxazole and antipyretics

A higher proportion of patients without malaria (defined as having a negative PCR result for malaria) were treated with antimalarial drugs in the control arm than in the mRDT arm (Table 3) (87.6% vs. 10.0%, *p* < 0.001). This major reduction in treating non-malaria patients with antimalarials was the main reason for the improvement in appropriate treatment.

Amongst the 42 cases of confirmed *P. falciparum* malaria in the intervention arm, only 28.6% (*n* = 12) received ACT. Of the 30 patients who did not receive ACT, 21 were missed by CHWs being guided by the mRDT result (false negative results). Of the 9 who were correctly diagnosed as *P. falciparum* positive by the RDT, 3 received CQ and 6 received no antimalarial drugs. ACTs were not available to the control arm as per local policy for treating unconfirmed malaria. However, in this arm, of 44 falciparum cases detected by PCR, 4 received no antimalarial drug, 34 received CQ monotherapy and 6 received SP and CQ. The net effect of introducing mRDTs was therefore a lower proportion of patients with falciparum malaria being given an antimalarial.

For vivax in the control arm, most patients were treated presumptively with CQ. A higher proportion of patients with PCR-confirmed *P. vivax* received CQ (the first-line treatment) in the control arm than in the mRDT arm (85.9% (*n* = 168) vs. 45.1% (*n* = 70), *p* < 0.001).

Amongst those with no malaria (PCR negative), 67.2% in the mRDT arm received the antibiotic co-trimoxazole, supplied to CHWs to treat pneumonia, compared to 35.0% in the control arm (*p* = 0.012). Antipyretics were prescribed to 846 (78.8%) in the control arm and 948 (84.8%) in the intervention arm (*p* < 0.001).

### CHW prescription decisions by RDT result

Analysis of CHW response to the RDT result shows that 826/950 (87.0%) of those with a negative mRDT result were not prescribed an antimalarial drug. In 117/149 (78.5%) of cases where a single line (pan line) (signifying vivax malaria) was recorded, CQ was prescribed as per national guidelines, and in a further 10 patients (6%) another antimalarial or combination effective against vivax was prescribed. Where two lines (Pf and pan) were recorded (identifying *P. falciparum*) 25/38 (66%) were prescribed SP + AS.

### Effect of age

A further analysis was undertaken restricted to those aged 5 years and below or above 5 years, comparing the primary outcome in each age band. For those under 5 years old, 64/82 (91.4%) in the intervention group and 6/46 (8.7%, *p* < 0.001) in the control group received appropriate treatment. In those over 5 years old, the relative proportions were 753/1001 (80.8%, intervention) and 179/1007 (19.2%, *p* < 0.001).

### Accuracy of mRDTs

Overall sensitivity under these operational conditions against the PCR result was low and specificity was acceptable for both pan-specific and falciparum-specific detection (Table 4). Sensitivity of the pan-specific tests under operational conditions was very low in the low-endemic northern province (2.1%, 95% CI: 0.1–11.3) and, although higher, was less than expected in the highly endemic eastern province (62.5%, 95% CI: 45.8–77.3).

**Table 4:** Sensitivity and specificity of mRDTs used in low and high transmission areas judged against PCR

	Sensitivity *n*/*N* (%, 95% CI)	Specificity *n*/*N* (%, 95% CI)
Pan-specific^a^		
Overall	122/225 (54.2, 47.5–60.9)	891/974 (91.5, 89.5–93.2)
Low transmission	1/47 (2.1, 0.1–11.3)	418/419 (99.8, 98.7–100)
High transmission	121/178 (68.0, 60.6–74.8)	473/555 (85.2, 82.0–88.1)
Pf-specific		
Overall	25/47 (53.2, 38.1–67.9)	1097/1133 (96.8, 95.6–97.8)
Low transmission	0/7	–
High transmission	25/40 (62.5, 45.8–77.3)	645/681 (94.7, 92.8–96.3)

^a^Detects any *Plasmodium* species including *P. vivax* and *P. falciparum*

To assess the possible causes of low sensitivity of the tests in detecting *P. falciparum*, we undertook an exploratory analysis to test the hypothesis that low parasite density infections (common in low endemic settings) may have led to a high rate of false negative mRDT results when interpreted by CHWs with standard training. We used the results from the qPCR analysis to approximate parasite density. The quantitation cycle (Cq) number for *P. falciparum* positive patients with mRDT results (i.e. in the intervention arm) were divided into a binary variable around the median. Those with a low Cq number had a higher copy number, indicating a higher parasite density. Those with Cq lower than the median (higher concentration of parasite DNA) had 17.4% (4/23) false negative results vs. those with a higher Cq (lower concentration of parasite DNA) having 78.3% (18/23) false negative results (Fisher’s exact test, *p* < 0.001), suggesting that the low operational sensitivity when RDTs were interpreted by CHWs was mainly due to poor test sensitivity at low parasite densities.

### Diagnosis, treatment and referral practices

We analysed the actions taken by the CHWs, including their final diagnosis, treatment and referral practices. In the intervention mRDT arm, CHWs diagnosed malaria in 249/1199 patients (20.8%). Of these, 86.6% had a positive mRDT result (either pan-line, or Pf/pan-line) with the remainder having a negative test result. In the mRDT arm, the most common alternative diagnoses to malaria as recorded by CHWs were either the common cold (*n* = 631, 52.6%) or pneumonia (*n* = 538, 44.9%). This contrasted with the 1180/1201 (98.3%) diagnosed with malaria in the control arm and is consistent with the higher use of co-trimoxazole in the mRDT arm.

There was no difference in the proportion of patients referred from the village health post to the clinic comparing the intervention arm and the control arm (34.6% vs. 26.4%; *p* = 0.116), Table 3.

## Discussion

This trial in moderate and low endemic areas of Afghanistan, typical in both epidemiology and healthcare provision to much of low-resource south Asia, showed that provision of mRDTs to CHWs together with practical training on correct use led to a complex picture. Targeting of antimalarials (the primary outcome) improved significantly, mediated by much lower rates of prescribing antimalarials to patients without malaria parasites. This useful outcome was offset by a significant reduction in prescribing antimalarials to those who did have malaria, both vivax and falciparum. ACTs were given to only a quarter of cases of falciparum malaria in the intervention arm. Antibiotic prescribing increased in the intervention arm, possibly indicating a switch from overprescribing antimalarials to overprescribing antibiotics.

In comparison to PCR, the operational sensitivity of WHO-approved mRDTs when used by CHWs was poor, possibly because of infections with low parasite density, which are common in low-transmission settings. Limited laboratory testing has suggested that, in common with many other tests, whilst the mRDTs used in this study have good sensitivity for vivax malaria at high parasitaemias, it may decrease rapidly (to around 50% at low parasitaemias) [18]. However, the prescribing practices of CHWs were highly concordant with RDT result, suggesting that if tests appropriate to the local setting were available, there would be a substantial positive effect on prescribing of antimalarials. In African settings CHWs have proved willing to adapt practice to RDTs [19, 20]. CHWs start with a history of fever in considering malaria; in the absence of either clear alternative diagnoses, or a test, malaria is unlikely but almost impossible to exclude.

WHO currently recommends diagnostic testing with WHO-approved RDTs prior to prescribing antimalarials. Trials in Africa including those in the formal secondary setting [21], district health posts (similar to this setting) [22] and private sector providers [23] support this approach, although health worker adherence to test results is variable [6, 24]. Translating this to the much lower incidence settings where most malaria is vivax and parasite density will be lower should be undertaken with caution in the absence of relevant data. This trial supports the principle that CHWs will change prescribing behaviour and improve targeting in acute febrile illness with mRDTs and training, but raises significant practical concerns. In particular, without mRDTs with greater operational sensitivity in this epidemiological setting, the risks of missing true cases of malaria, including falciparum, are non-trivial. In Africa the risks of missing falciparum malaria are emphasised early in training, and studies show it is very rare for test positive cases not to be prescribed antimalarials [13]. In south Asia the true risk of malaria is lower, and it is possible that this drives behaviour [25].

The introduction of mRDTs, whilst reducing the overuse of antimalarial drugs, had the effect of increasing the use of antibiotics (co-trimoxazole) in malaria negative cases; this finding is similar to operational studies in Africa [5, 22], and recent studies suggest that this is a widespread concern in the context of rising antibiotic resistance as a consequence of overuse [26]. A previous individually randomised trial at a higher level of the health system suggested that the better trained personnel in those centres are more likely to diagnose malaria with mRDTs [11], but also tend to switch from overuse of antimalarials to higher use of antibiotics. Interventions are required which improve prescribing practices in patients with non-severe fever.

The study was designed to assess the effectiveness of the mRDT intervention under field operational conditions within a widely accessed part of the health service in malaria endemic areas of Afghanistan, and has the advantages and limitations of operational studies. The cluster design allowed a robust measurement of the effect of mRDTs on prescription of antimalarial drugs by CHWs and is accepted as a more reliable approach than individual patient randomised trials conducted in clinics, because clinicians influence one another. Training followed the approved national guidelines for the use of mRDTs, which closely match the WHO standard guidelines for training and use of mRDTs, and so is generalisable. The clinics chosen were representative of most of Afghanistan’s health service, and were similar to many CHW systems in low-resource south Asia. The main findings and conclusions should therefore be applicable to other countries in the region. The main limitations were imposed by having to work through relatively unskilled CHWs in a relatively unstable area. In particular it was not realistic to get blood slides made to supplement PCR data and/or to follow patients up into the community. PCR is significantly more sensitive than microscopy, but in a low-transmission setting it is unlikely that a PCR positive malaria case would not be clinically relevant [27]. If microscopy could have been reliably used as a gold standard in this clinical setting, then sensitivity of the test may have been reportedly higher. Lastly, a cost-effectiveness analysis was not part of this study, but it is an important consideration for policy decisions driving the placement of mRDTs at the facility and/or community level [28].

Use of mRDTs by CHWs has a potential advantage of improving surveillance of disease based on parasitological diagnosis. This is particularly relevant to malaria epidemiology in Afghanistan, which is heterogeneous. In the north of the country there is very little transmission, and elimination is being actively pursued. This requires an ability to detect and respond to outbreaks with preventative interventions such as insecticide-treated nets. However, the performance of the mRDTs at the community level may reduce the attractiveness of this tool in favour of more sensitive tests. In the east of the country , the malaria programme is still in the control phase, where mRDTs could provide improved surveillance and targeting of preventative measures.

The increase in antibiotic use in the intervention arm is potentially a concern. The World Health Assembly and UN General Assembly have recently highlighted the serious risk that is posed by antibiotic resistance. Overuse of antibiotics in south Asia is driving substantial antibiotic resistance [29]. If one of the effects of mRDTs is to lead to a switch from overprescribing of antimalarial drugs to overprescription of antibiotics, this would be a concerning unintended consequence.

## Conclusions

In low prevalence areas such as Afghanistan, mRDTs for malaria have at least three potential roles: to identify the relatively uncommon cases of true malaria, especially falciparum malaria; to provide epidemiological surveillance data in areas considering elimination; and to reassure prescribers that not prescribing an antimalarial is reasonable. The main advantage of mRDTs at the community level is to reduce the overuse of antimalarial drugs in malaria negative patients. However, currently available mRDTs may miss a substantial proportion of cases of malaria. Although using mRDTs led to reductions in the number of inappropriate doses of antimalarials, mRDTs may also lead to overprescription of antibiotics. Any potential role for improving surveillance should consider the operational sensitivity of mRDTs, which may be too low for detecting some malaria infections; higher sensitivity tests may be useful in these settings. The risks identified in this study need to be balanced against the advantages of rolling out mRDTs amongst CHWs.

## Additional file

Additional file 1: ACT Consortium, Afghanistan. Cluster Randomised Trial (study 1.2, CRT). (DOCX 484 kb)

## Abbreviations

[m]RDT: [Malaria] rapid diagnostic test
ACT: Artemisinin combination therapy
CHW: Community health worker
PCR: Polymerase chain reaction
