# Supplementary material for: Use of malaria rapid diagnostic tests by community health workers in Afghanistan: cluster randomised trial
Source: BMC Med. 2017 Jul 7;15:124. doi: 10.1186/s12916-017-0891-8 (PMC5501368; doi:10.1186/s12916-017-0891-8)
Supplement: Additional file 1: — ACT Consortium, Afghanistan. Cluster Randomised Trial (study 1.2, CRT). (DOCX 484 kb) [file 12916_2017_891_MOESM1_ESM.docx]

**ACT Consortium, Afghanistan. Cluster Randomised Trial (study 1.2, CRT):**

**Effectiveness of community level deployment of rapid diagnostic tests for malaria in Afghanistan: Cluster Randomised Trial.**

**Outline Protocol:**

**FINAL DRAFT**

**September 2011**

**DRAFT: Not for circulation**

# Table of Contents:

[1. Background and rationale: 5](#_Toc303771208)

[1.1. Context of the study: 5](#_Toc303771209)

[1.2. Scientific Rationale: 5](#_Toc303771210)

[2. Study Design and Methods: 7](#_Toc303771211)

[2.1. Study Objectives: 7](#_Toc303771212)

[2.2. Study Sites and Patient Enrolment: 7](#_Toc303771213)

*[2.2.2.](#_Toc303771214)**[Number of Health-posts per clinic:](#_Toc303771214)* [7](#_Toc303771214)

[2.3. Pre-study CHW data: 9](#_Toc303771215)

[2.4. Post-intervention evaluation of CHW Practice: 9](#_Toc303771216)

[2.5. Cluster Randomised Study. 11](#_Toc303771217)

[2.5.1. Trial Arms: 11](#_Toc303771219)

[2.5.2. Randomisation for Phase I: 13](#_Toc303771223)

[2.5.3. Balance criteria: 13](#_Toc303771224)

[2.5.4. Randomisation Methods: 13](#_Toc303771225)

[2.5.5. Results of randomisation 15](#_Toc303771230)

[2.5.6. Trial Profile: 17](#_Toc303771233)

[2.5.7. Case definition and patient enrolment: 18](#_Toc303771234)

[2.6. Outcomes: 19](#_Toc303771236)

[2.6.1. Trial Primary Outcome: 19](#_Toc303771237)

[2.6.2. Trial Secondary Outcomes: 19](#_Toc303771238)

[2.6.3. Evaluation Criteria: 20](#_Toc303771239)

[2.7. Outcome Measurement and Recording: 20](#_Toc303771240)

[2.7.1. Outcome Measurement: 20](#_Toc303771241)

[2.7.2. Data recording: 21](#_Toc303771242)

[2.8. Sample Size and Statistical Methods: 22](#_Toc303771243)

[2.8.1. Sample Size Assumptions: 22](#_Toc303771244)

[2.8.2. Number of consultations per clinic and health post: 23](#_Toc303771245)

[2.8.3. Sample size: 23](#_Toc303771246)

[2.8.4. Statistical Methods: 24](#_Toc303771251)

[2.9. Laboratory Methods: 25](#_Toc303771253)

[2.9.1. RDT Selection: 25](#_Toc303771255)

[2.9.2. Sample Collection: 25](#_Toc303771256)

[2.9.3. RDT Accuracy: 25](#_Toc303771257)

[2.9.4. PCR Evaluation: 25](#_Toc303771258)

[2.10. Ethical Issues: 26](#_Toc303771259)

[2.10.1. Informed consent: 26](#_Toc303771260)

[2.10.2. Confidentiality: 26](#_Toc303771261)

[2.10.3. Risks and potential adverse events: 26](#_Toc303771262)

[3. Programme Implementation: 27](#_Toc303771263)

[3.1. Staff structure: 27](#_Toc303771264)

[3.2. Staff roles: 27](#_Toc303771265)

[3.3. Staff training: 28](#_Toc303771266)

[3.4. Implementation Plan: 30](#_Toc303771273)

[References 31](#_Toc303771274)

[Annex 1: CHW enrolment and activity form: 33](#_Toc303771275)

[Annex 2: Revised IMCI algorithm for treatment of fever at community level: 40](#_Toc303771276)

[Annex 3: Evaluation indicators for National CBMM strategy (benchmarks for Arm 2). 41](#_Toc303771277)

[Annex 4: CHW Data collection form. 43](#_Toc303771278)

[Annex 5: Clinic Patient Tracking Form: 45](#_Toc303771279)

[Annex 6: Information and consent form for patients enrolled by CHWs. 47](#_Toc303771280)

[Annex 7: Information and consent form for patients enrolled at clinics. 49](#_Toc303771281)

[Annex 8: Consent form for CHW Participation: 51](#_Toc303771282)

# Principle and co-Investigators:

Principle Investigators:

LSHTM: Mark Rowland

For LSHTM in Afghanistan: Toby Leslie (Co-PI), Amy Mikhail (Project Manager)

Co-investigators:

LSHTM: Bonnie Cundill, Clare Chandler, Kristian Hansen, Shunmay Yeung, Chris Whitty.

HPRO: Ismail Mayan, Rohullah Zekria, Habib.

HealthNet TPO: Mohammed Nader, Mohammed Anwar Hasan Zai.

Merlin: Sayed Habib Bakhtash, Sayed Hussein Hashimi

NMLCP: Mohammed Sami

# Background and rationale:

## Context of the study:

This document describes an updated protocol for study 1.2 in the original ACT Consortium Protocol (“An examination of ACT strategy in south-central Asia on falciparum malaria in a context where vivax is the major species”). The ACTc Programme in Afghanistan has been running since 2008 in two provinces, Kunduz in the North (run by Merlin) and Nangahar in the East (run by HealthNet TPO). The study aims to examine the effectiveness of different diagnostic strategies for improving targeting of antimalarial drugs.

Since the drafting of the original concept for study 1.2 (Cluster randomised study), three significant policy and operational changes have occurred in the study areas.

- Implementation of new microscopy centres (in Kunduz province).
- Large scale distribution of ITNs in the study provinces
- The planned implementation of a large national programme to scale up access to diagnostics at community and clinic levels.

These changes have had a significant effect on the policy and operational setting of the project and have changed the overall strategy of this stage of the programme. Firstly, additional clinics both in the study areas and elsewhere will be provided with microscopy and/or RDTs for diagnostics and ITNs will have been widely distributed in the study areas including most households. More significantly, a program to deliver RDTs for malaria diagnosis through 150 health posts staffed by Community Health Workers in endemic areas began this year.

The ACTc programme has examined the effectiveness of RDTs for targeting antimalarial drugs at the primary clinic level by measuring the accuracy of treatment under different diagnostic conditions. Microscopic (both established and new), clinical (i.e. non-parasitological) diagnosis and RDTs have been compared using a randomised trial design. Results of these surveys are forthcoming and will provide data on the implementation of diagnostics at the clinic level. In view of the forthcoming policy change to deliver RDTs at the community level through networks of community health workers, this protocol describes a plan for evaluating the effectiveness of RDT delivery at community level in targeting of antimalarial drugs.

## Scientific Rationale:

Targeting of antimalarial drugs is an important priority for delivery of effective treatments and requires parasitological diagnosis of malaria parasites and prescription of appropriate treatment. In areas where cases result from two or more species (typically *Plasmodium falciparum* and *P. vivax*) treatments for the two species differ. Because falciparum malaria has developed resistance to chloroquine, which remains effective against vivax malaria, identification of the species of malaria is vital for targeting ACTs.

Most malaria in Afghanistan is caused by vivax, which is a minority (although significant) cause of febrile illness. A few (around 5-10%) of cases are caused by falciparum malaria, which is treated with more expensive artemisinin combination therapy drugs (ACT). Identifying these few cases of falciparum and targeting the more expensive drugs at the few cases that occur is the major challenge. Mistreatment of falciparum as vivax (i.e. with chloroquine) could lead to poor treatment outcomes, where vivax treated as falciparum (i.e. with the more expensive ACT drugs) wastes expensive drugs. This places great importance on access to accurate diagnosis that can distinguish between the species.

Current research conducted at clinic basic health centre level shows that when parasitological diagnosis (using either RDTs or microscopy) is implemented at the clinic level, there is an improvement in treatment accuracy (i.e. the proportion of patients who are correctly treated) when compared to clinical diagnosis only (i.e. where there is no identification of parasites). However, despite this improvement around 50% of patients who are negative for malaria parasites are still treated with antimalarial drugs. The research conclusions show that improving the targeting of drugs requires not only the implementation of parasitological diagnosis, but also a change in prescriber behaviour. The over treatment for malaria, despite negative laboratory results, wastes drugs, reduces the cost-effectiveness of the diagnostic method, and (most importantly) leads to mistreatment of other causes of fever.

While the rollout of RDTs at clinic level has some evidence base (in part, from the previous ACTc studies), there is little available data on the effectiveness with which this intervention can be provided at community level through CHWs. The data that does exist locally is based on two studies, one conducted in 2006^[[1]](#footnote-1)^ and one ongoing pilot programme^[[2]](#footnote-2)^. Evidence from the study and from the pilot programme suggests that RDTs are not utilised effectively by CHWs. In the study, high rates of invalid tests were reported due to user error, and in both programmes, RDTs are incorrectly applied to patient’s symptoms. The results are that very large numbers of RDTs are used, and very few cases of malaria are adequately detected. The effect of RDTs on treatment decisions and accuracy was not noted by either study.

Use of RDTs at community level will also have an effect on current guidelines for management of febrile illness and diseases of children. The current community IMCI guidelines for Afghanistan (and most of Asia) do not include RDTs in the differential diagnosis of childhood diseases in the community. The current guidelines favour a blanket approach to treatment of “suspected malaria” where there is no other obvious cause of illness and the patient is in an endemic area. Changing these guidelines requires an evidence based approach to assess both the clinical and economic aspects of the intervention – something which has thus far not been conducted despite the trend in programming which has now started to deploy RDTs much more widely.

This study aims to provide evidence on the effectiveness of RDTs that have been deployed at community level by measuring the accuracy of the tests themselves, and the accuracy with which treatment is provided to malaria positive and malaria negative cases.

# Study Design and Methods:

## Study Objectives:

Primary Objective: To assess the effectiveness of deployment of malaria rapid diagnostic tests by Community Health Workers for improving access and targeting of antimalarial drugs in Afghanistan using a cluster randomised intervention study in two phases.

Secondary Objectives:

- To assess the current role of Community Health Workers in the treatment of fever (as defined by the CHW).
- To assess the effect of RDT implementation amongst community health workers on accuracy and timing of treatment in patients presenting with fever.
- To assess the effect of RDTs on the treatment of children presenting with fever at community level.
- To assess a package of interventions that aims to improve treatment targeting by community health workers.
- To examine the cost effectiveness of each intervention.
- To examine the effect of RDT on provider and user behaviour using qualitative methods.
- To make recommendations for policies which can improve community based health programmes (for example, IMCI).

## Study Sites and Patient Enrolment:

- - 1. *Clinics:* The study will be conducted using 22 clinics which have previously been used for ACT Consortium research. Twelve clinics are situated in Nanagahar Province (East Region) and ten clinics are based in Kunduz Province (North Region). The study areas have varying malaria endemicity, with higher incidence in the East Region. All clinics have been included as study sites for the previous phases of the project and are included in this study as a representative sample of typical clinics in different malaria endemic areas. The clinics are all primary care clinics (Basic Health Centre or Comprehensive Health Centre). They are staffed by a clinic doctor, who acts as the manager, and other staff (for example, nurses or midwives) who occasionally run the clinic when the doctor is away from post. Each centre also has a community health supervisor, who is responsible for the community health workers attached to each clinic.

## *Number of Health-posts per clinic:* Amongst the 22 clinics, there are 196 health posts, with 65 in Kunduz and 142 in Nangahar (table).

| **Province/clinic** | **Number of Health Posts** |
| --- | --- |
| **Kunduz** | **65** |
| Ajighan | 6 |
| AngorBagh | 7 |
| ArbabRamazani | 2 |
| BolaQachi | 5 |
| CharSari | 6 |
| Khan Abad | 20 |
| KhwajaGhaltan | 8 |
| Madrasa | 3 |
| Nick Pay | 3 |
| SandoqSai | 5 |
| **Nangarhar** | **142** |
| Baro | 8 |
| Batikot | 21 |
| Behsood | 16 |
| Chardhi | 6 |
| GardiGhos | 6 |
| Khewa | 12 |
| LandaBuch | 10 |
| MamandDara | 11 |
| NajmulQura | 6 |
| Rodat | 13 |
| SangarSarai | 18 |
| Shegay | 15 |
| **Grand Total** | **207** |

Each health-post has two staff – one female and one male CHW who will be enrolling patients into the study.

- - 1. *Health System at Community level:* The clinics act as a base for community based interventions. Community interventions are provided through a network of community health workers (CHWs) (for which community midwives are also included in some areas). The CHWs are administratively attached to a single clinic and are supervised by the Community Health Supervisor (CHS). There are between 3 and 10 CHWs attached to each clinic, depending on the catchment area or population of the clinics. CHWs operate within their communities, from their homes (called Health Posts). CHWs are volunteers who are selected by the communities they serve. They have numerous roles within the health system, including:
- Acute care and first-aid (including IMCI)
- Delivery of EPI and polio vaccine
- Delivery of insecticide treated nets
- Health education and awareness
- Attendance at trainings
- Health Information and Management data reporting
- Midwifery services (community midwives)

Typically, CHWs have had general training as well as multiple other trainings conducted by individual programmes (for example, the National Malaria Control Programme, EPI). They may be semiliterate, or illiterate, and have varying levels of training.

## Pre-study CHW data:

*Pre-study interviews:*

Data collected from all CHWs using a basic information form filled out at start of pre-study period (annex 1 for form):

- Personal data
- HP name/number
- Educational background / literacy
- Work history (last 3 years?)
- Current job other than a CHW
- GPS coordinates of the HP (if feasible), if not estimated distance to the mother-clinic.
- Length of time living in the village / area
- Socio-economic background / status

At this stage, the CHW will be assigned a CHW number (e.g. cNBA/01). Newly enrolled CHWs (i.e. for replacements) will be assigned the next sequential number on the list (e.g. cNBA/07).

## Post-intervention evaluation of CHW Practice:

At the end of phase 1 (and repeated at the end of phase 2), an evaluation will examine the current practice of CHWs in diagnosis, treatment and referral of malaria cases. The objective is to describe differences in practice between the two arms of the trial.

Secondly the post-intervention study will use structured observational studies (using a checklist), semi-structured interviews with the CHWs, their community health supervisor (CHS) and others involved in CHW task scheduling, and review of programme records on the activities of CHWs who will be included in the study. This will provide information on current practices, standards, quality and deficiencies (Annex 1).

The study will establish indicators and potential explanatory variables through interviews, observation and review of programme records, with the primary comparison being between the two intervention arms:

Review of Programme Records:

- Number of CHW activities per month for each HP attached to our trial clinics over the trial length.
- Type of activity (proportion health education, preventative, consultations)
- Age range of patients seen by CHWs
- Location of activities (travel log) – primary data spanning 1 typical month
- Number of consultations per week with reasons for each (e.g. malaria, URTI, distribution of condoms, etc)
- CHW supervisory visits by CHSs and visits by CHWs to CHSs (frequency, what tools are used during these visits, feedback mechanisms) – previous month (May 2011 – AFG Calendar)

Data collected from a sub-group for more detailed interviews, stratified by province, gender and clinic type (CHC vs. BHC) (Soc Sci Officers).

- Current CHW practice where malaria is suspected, using case studies and in depth interview techniques – primary interview in random subsample.
- Motivation for the job
- Time per activity data – pre and during the trial (at end of phase 1) over 1 week
- Knowledge of malaria, fever, IMCI, and blood safety
- Current CHW IMCI practice using case studies and in depth interviews
- Current CHW referral practice (under what clinical circumstances and how many per week)CHW drug stock (types, quantity, expiry dates, storage conditions)
- CHW re-stock procedure (via interview if it does not occur during observation)
- CHW job-aids and reporting templates (types provided, how they are used)
- CHW basic training (description of what is covered) and any further training given
- CHW literacy and pre-training education level
- CHW day job (any paid employment that is normally undertaken).
- CHW schedule (as prescribed by BPHS implementer and CHS)
- Motivation and personal factors influencing their roles as CHWs

The study will also examine supervisory and community structures by interview and focus groups with Community Health Supervisors and community members/leaders.

At the end of phase 1 and end of phase II of the trial (see next section), a series of social science assessments will be conducted.

In-depth interviews will be conducted with a purposive sample of CHWs, to be conducted after phase 1 of the trial.  The CHWs will be divided into groups according to how they responded to the trial; group A will be "controls" who dealt with the patient appropriately (selected for diagnosis, interpreted RDT, treated, referred) and other groups as needed who did not deal with the patient appropriately.  These groups will be defined by the "problem responses" of CHWs that come up in the data; for example if all CHWs select patients for diagnosis with an RDT correctly and refer correctly but do not treat according to the RDT results correctly, then the CHWs who did not treat correctly will form group B.  Approximately 5 CHWs will need to be interviewed from each group, for each arm.  Responses will be compared between arms.  Topic guides for these CHW IDIs will be developed as the trial progresses and information collected which can guide the development of the guides.

In-depth interviews to be conducted with a purposive selection of patients, during phase 1 of the trial. The interviews will be used to identify, in general, the patient perspectives of CHW services, what they use them for and when, as well as whether there has been any change in their perceptions of these services since the start of the trial (re introduction of RDTs etc).  For the latter question, patient IDIs from both arms will be compared.  Approximately 10 patients will be selected from each arm.  Again, topic guides will be developed during the trial.

Focus group discussions to be held with community leaders after phase 1 of the trial.  The purpose is as for patient IDIs above, to determine their perceptions of CHW services and note any changes since trial.  Approximately 3 community leaders will be selected per arm (i.e. 2 focus group discussions).  Again, topic guides will be developed during the trial.

## Cluster Randomised Study.

The main study will be a cluster randomised trial conducted in two phases. Clinics will initially be randomised to one of two arms in the trial and then subsequently, clinics will be randomised a second time to two additional trial arms.

### Trial Arms:

**Phase I:**

*Arm 1: Non-intervention arm:*

The non-intervention arm will be based on the existing clinical diagnosis, pre-referral treatment and referral to a clinic in areas where RDTs have not been deployed at CHW level. Arm 1 CHWs will receive refresher training according to the National Strategy for CBMM which includes the current IMCI guidelines.

Vs:

*Arm 2: RDT Arm*

Provision of community level diagnostics by rolling out RDTs to CHWs through the national strategy for Community based Management of Malaria. The arm has been designed exactly as the current National guidelines for RDT implementation..The arm will include:

- Clinic level microscopy or RDT and CHWs using RDTs to provide parasitological diagnosis to diagnose, treat and/or refer patients. Under this scheme, CHWs will be provided with CQ, SP and artesunate (AS). Depending on the diagnostic result, they prescribe according to the National Treatment Guidelines (CQ for confirmed vivax, SP and AS for confirmed falciparum, and no antimalarial for a negative result).
- CHW training will be conducted exactly as described in the CBMM strategy, using a “trickle down approach” where Training of Trainers takes place at the central level for provincial trainers of local NGOS who run the health service. These, in turn, run workshops for training CHSs and CHWs (see section 2.2 for pre-evaluation of training and roll-out). Participants will receive training to enforce revised IMCI guidelines, taken from the CBMM strategy document, which include an RDT in the algorithm for fever (see annex 2).
- There are no plans for additional support or refresher training, and no plans for quality control and assurance contained in the National Plan.
- CHWs are not equipped with suitable storage facilities for the RDTs, for example evaporative coolers.
- Monitoring and evaluation takes place according to the draft strategy.

The second phase of the trial will be initiated in Feb 2012. The second phase of the trial will re-randomise clinics to either the RDT arm (as in arm 2, above) and a third arm (arm 3). The third arm will answer the question: “how can the performance of RDTs at community level be enhanced?”. The same set of outcomes will be used to measure the relative effectiveness of each approach. A revised random allocation using the same methods will be conducted for the Phase II randomisation.

**Phase II**

*Arm 2: RDT arm (as above)*

*Vs.*

*Arm 3: Extended Programme Rollout of RDTs:* The intervention arm will consist of an extended programme of implementation. The intervention is designed with the aim of providing a best-practice model for rollout of RDTs at community level. The intervention arm will consist of a range of supportive interventions alongside the RDT roll-out:

- Clinic level microscopy or RDT and CHWs using RDTs to provide parasitological diagnosis to diagnose, treat and/or refer patients. Under this scheme, CHWs will be provided with CQ, SP and artesunate (AS). Depending on the diagnostic result, they prescribe according to the National Treatment Guidelines (CQ for confirmed vivax, SP and AS for confirmed falciparum, and no antimalarial for a negative result).
- CHW training will be conducted by dedicated trainers. Cadres of CHWs for each clinic will be trained which will include several modules (basic febrile illness management and additional training on IMCI and use of RDTs within the IMCI guidelines, clinical practice in RDTs, prescription of antimalarial drugs, blood safety and sharps disposal, treatment of RDT negative patients) as well as practical training.
- CHWs will be provided with regular support to review their application of RDTs using case-studies.
- RDTs and CHWs will be actively involved in improved quality assurance and quality control procedures
- CHWs will be issued with evaporative coolers to store RDTs
- Other supportive interventions will be considered following the results of the initial survey, interim analysis of the social science components and phase 1 of the trial.

### Randomisation for Phase I:

In CRT with a small number of clusters (e.g. <15-20 per arm) and strong between-cluster correlation there is an increased chance of an imbalance on one or more covariates across the intervention arms. If these covariates are also expected to be important correlates of the primary outcome then this can influence the interpretation of the study results. Although imbalances may be adjusted for in cluster-level analysis there is some inadequacy in this approach and therefore Raab (2001) and Moulton (2004) suggest constraining the randomisation through restriction to those allocations that meet specified criteria, to achieve balance at the start. They suggest constraining enough so as to ensure marginal balance on relevant covariates.

### Balance criteria:

The following variables that are expected to be important correlates of the primary outcome and on which we wish to achieve balance were based on a data collected in 2009 in the same study clusters and over the same study period i.e. July to December.

1. Harmonic mean. For a given number of clusters and overall sample size maximum statistical efficiency is obtained if an equal number of patients per cluster are selected. The total sample size of 1500 across 22 clusters would require an *arithmetic* mean of 68 patients per cluster. However, due to the huge variability in the proportion of patients seen with suspected diarrhoea, URTI and malaria (when CHWs are likely to use RDTs) per cluster this approach was not seen as appropriate for several reasons including time to reach sample size quota, and cluster representation. Hence the sample size was weighted according to the cluster proportion of patients seen with suspected diarrhoea, URTI and malaria to achieve a *harmonic* mean of 68 patients per cluster in each arm. Since the sample size calculation was done before randomisation the overall harmonic mean (for all clusters) was used but this does not guarantee a harmonic mean of 68 per arm and therefore this was included as a restriction factor in the randomisation.
2. Total sample size. The precision and power of a study are usually maximised when sample sizes are similar in the treatment arms. In CRTs this means that the number of clusters as well as the number of individuals should be similar across treatment arms. Therefore, due to the variability in sample size per cluster this was also added as a restriction factor.
3. Number of health posts. There is a substantial amount of variation in the number of health posts per cluster from 2 to 26. Clusters (clinics) with bigger catchment populations have more health posts and therefore by balancing on this factor and equal sample size per arm will be achieved.

### Randomisation Methods:

### Number of unrestricted allocations

The study is a stratified cluster randomised trial with a total of 22 clusters.

In Kunduz the total number of ways of allocating the 10 clusters to the two intervention groups, assuming equal number of clusters per arm:

$$\binom{10}{5}\binom{5}{5}=252$$

In Nangahar the total number of ways of allocating the 12 clusters to the two intervention groups, assuming equal number of clusters per arm

$$\binom{12}{6}\binom{6}{6}=924$$

Therefore in total there are: 252 x 924 = 232,848 possible allocations of 22 clusters into the 2 study arms after stratification.

### Exploring individual restriction factors

A compute program (R statistical environment) enumerated each of these allocations to identify which satisfy the balance criteria.

For each balance criteria, restriction factors^^[[3]](#footnote-3)^^ were estimated for various tolerance thresholds for the measure of imbalance. Table 1 presents the results for individual balance criteria in terms of the proportion of acceptable allocations and the corresponding restriction factor.

Table 1: Proportion of acceptable allocations and restriction factors for various tolerance thresholds

| **Criteria** | **Tolerance threshold** | **Proportion acceptable** | **Restriction factor** |
| --- | --- | --- | --- |
| Total sample size | 50 | 0.119 | 88.1% |
|  | 60 | 0.142 | 85.8% |
|  | 70 | 0.165 | 83.5% |
|  | 80 | 0.188 | 81.2% |
|  | 90 | 0.210 | 79.0% |
|  | 100 | 0.232 | 76.8% |
|  | 150 | 0.342 | 65.8% |
|  | 200 | 0.445 | 55.5% |
| Harmonic Mean range | 67-69 | 0.032 | 96.8% |
|  | 66-70 | 0.085 | 91.5% |
|  | 65-71 | 0.141 | 85.9% |
|  | 64-72 | 0.196 | 80.4% |
|  | 63-73 | 0.250 | 75.0% |
| Number of health posts | 5 | 0.120 | 88.0% |
|  | 6 or 7 | 0.168 | 83.2% |
|  | 8 or 9 | 0.215 | 78.5% |
|  | 10 | 0.263 | 73.7% |
|  | 15 | 0.356 | 64.4% |
|  | 20 | 0.490 | 51.0% |

### Exploring combinations of thresholds

Combinations of thresholds were examined and the final choice was based on achieving acceptable balance between the study arms and maintaining an unbiased and valid design. As described by Moulton (2004) a design is biased if there is any difference across the clusters in their probability of allocation to any given treatment. A randomised design is said to be valid if every pair of clusters has the same probability of being allocated to the same treatment. An unbiased design is unlikely to occur in practice but the validity is more questionable when conducting a constrained, or restricted, randomisation. If the design is not valid there is a risk that the Type I error changes from its nominal value of 0.05.

### Final combination of balance criteria

The final choice included the following restrictions:

1. Total sample size per arm differing by no more than 50
2. Harmonic mean in each cluster being within the range 65 to 71
3. The number of health posts per arm differing by no more than 10 health posts

### Results of randomisation

The final choice of restriction criteria resulted in 1598 acceptable allocations from which one was randomly chosen. Table 2 and **Error! Reference source not found.** summarise the allocation process.

6 clinics (clusters) have been allocated to each study arm in the Nangahar stratum, and 5 per arm in Kunduz stratum. Study arm 0 denotes no RDT, and study arm 1 denotes RDT (intervention) – see next section.

Table 2: Results of intervention allocation process

| **Kunduz** | | |  | **Nangahar** | | |
| --- | --- | --- | --- | --- | --- | --- |
| **Cluster** | **Clinic** | **Study arm** |  | **Cluster** | **Clinic** | **Study arm** |
| 1 | Ajighan | 0 |  | 11 | Baro | 0 |
| 2 | AngorBagh | 0 |  | 12 | Batikot | 1 |
| 3 | ArbabRamazani | 0 |  | 13 | Behsood | 0 |
| 4 | BolaQachi | 1 |  | 14 | Chardhi | 1 |
| 5 | CharSari | 1 |  | 15 | GardiGhos | 1 |
| 6 | Khan Abad | 1 |  | 16 | Khewa | 0 |
| 7 | KhwajaGhaltan | 0 |  | 17 | LandaBuch | 0 |
| 8 | Madrasa | 1 |  | 18 | MamandDara | 1 |
| 9 | Nick Pay | 1 |  | 19 | NajmulQura | 1 |
| 10 | SandoqSai | 0 |  | 20 | Rodat | 0 |
|  |  |  |  | 21 | SangarSarai | 1 |
|  |  |  |  | 22 | Shegay | 0 |

Table 3: Summary of clusters allocated to study arms in Phase 1

| **Arm 0 (no RDT)** | **Arm 2 (RDT)** |
| --- | --- |
| Ajighan | Batikot |
| AngorBagh | BolaQachi |
| ArbabRamazani | Chardhi |
| Baro | CharSari |
| Behsood | GardiGhos |
| Khewa | Khan Abad |
| KhwajaGhaltan | Madrasa |
| LandaBuch | MamandDara |
| Rodat | NajmulQura |
| SandoqSai | Nick Pay |
| Shegay | SangarSarai |

### Baseline characteristics by study arm

It can be seen from **Table 4** that given the above restricted randomisation we achieved a suitable balance on those factors included in the randomisation. A baseline table showing all individual and cluster-level covariates would also be interesting to include in a paper to examine the balance on factors not included in the randomisation.

**Table 4: Examining the balance after randomisation**

|  | **No RDT (n=11)** | **RDT (n=11)** |
| --- | --- | --- |
| Total sample size | 1280 | 1262 |
|  |  |  |
| Harmonic mean number of patients per cluster | 66.03 | 69.28 |
|  |  |  |
| Number of health posts | 111 | 109 |

### Validation matrix

To assess the validity of the design for the combination of thresholds a matrix was made. The columns and rows of the validation matrix represent each of the 22 clusters. The elements of this matrix are the proportion of times each pair of clusters is allocated to the same group in the 1598 acceptable allocations i.e. the probability that the ith cluster is being allocated to the same intervention group as the jth cluster. The matrix was then examined for under- and over-represented pairs that would highlight any potential causes for concern in the randomisation.

The validation matrix for the final choice of restriction criteria showed that, with the exception of two pairs (3,14) and (9,20), the matrix elements range from 20% to 70% indicating under- and over-representation of a few clusters but the majority were around 50% illustrating a valid randomisation. Inspection of the data showed no obvious reasons for the two pairs (3,14) and (9,20) only being together in 0.1% of the allocations, and could not be increased by loosening the thresholds. However, despite this there are still 159 possible allocations in which these two pairs of clusters can be allocated to the same study arm; hence it was concluded that this restriction criteria was valid.

### Trial Profile:

**Figure: Trial schematic for Phase 1.**

If the criteria for running phase 2 of the trial are met the 22 clinics will be re-randomised so that 50% of those clinics in arm 1 are randomised to arm 3 and 50% to arm 2, and 50% of those in arm 2 will be randomised to arm 3 with 50% remaining in arm 2 (figure 2).

**Figure 2: Phase 2 trial schematic.**

### Case definition and patient enrolment:

Patients will be drawn from the communities where the CHWs operate and the clinics which supervise and support those CHWs. Inclusion criteria will be:

- Any patient where the CHW* or clinician considers malaria in the diagnosis – either prescribing an antimalarial or would request a malaria test if available or referring for diagnosis of malaria elsewhere.
- Patient, or parent/guardian, gives informed consent to the study.

*any community health worker who consults with patients and prescribes treatment, and is administratively attached to one of the study clinics.

Exclusion criteria will be

- Patients with a diagnostic result from another facility.
- Patients referred on for diagnosis in the private sector.
- Patients who have signs of severe or complicated disease and are referred prior to giving any diagnosis using the interventions.

Patients can be enrolled during any consultation with a CHW no matter where it takes place. The consultation could be made at the health post, the patient’s residence, or even outside in the street or fields. The CHW will also use the standard national HMIS tool for recording all their consultations, which will also allow assessment of any changes to practice within the community by identifying changes in the number of each type of consultation (for example, community DOTS, health awareness, condom distribution, etc).

If the patient gives informed consent, the CHW will assign a unique patient number and note the patients information on a pro-forma pictorial record form (Patient Tracking Form, see section 2.6). The patient tracking form has been designed for semi literate and illiterate health workers and has been pretested and validation in field testing using CHWs in Nangahar province.

Patients will also be recruited in clinics during the study at clinic level. Those who present without visiting a CHW (i.e. have not been enrolled at community level and self refer) will be enrolled according to the inclusion and exclusion criteria. Patients who present at the clinic who have been enrolled at community level will also be enrolled if they present to the clinic and these will be linked to the consultation at community level though the use of the patient ID card - All patients enrolled at community level will be given a study patient ID card which will identify them as having been enrolled by a CHW and note their patient number from the community level enrolment.

## Outcomes:

The trial outcomes are presented below. In addition, the RDT arm (arm 2) will be subjected to an interim evaluation for which outcomes are also presented.

### Trial Primary Outcome:

The primary outcome of the study is:

- Proportion of patients (in)correctly treated by CHWs.

Correct treatment is defined as:

- - Treatment of PCR confirmed Pf with SP/AS.
  - Treatment of PCR confirmed Pv with CQ (regardless of accompaniment with SP or addition of primaquine).
  - No antimalarial for PCR confirmed negative diagnosis.

### Trial Secondary Outcomes:

- % of Pf cases treated with ACT.
- % of patients <10 years of age who are correctly treated (estimated at 40% of all patients)
- % of malaria negative patients receiving antimalarial drug
- % of malaria negative patients receiving antibiotics
- % of patients treated per policy

Per policy defined as:

- Suspected malaria which is PCR confirmed treated with CQ/SP
- Community based diagnosed Pf, subsequently confirmed as Pf treated with SP/AS
- Community based diagnosed Pv, subsequently confirmed as Pv treated with CQ
- No antimalarial given to negative cases (confirmed by PCR)
- Proportion of patients incorrectly treated in each arm, including data collected at the clinic level.
- Time to first treatment from onset of symptoms.
- Cost per patient correctly treated.
- Cost per patient treated per policy.
- Invalid rate, specificity and sensitivity of RDTs deployed at community level (against PCR).
- Number of patients attended by CHWs and type of attendance.
- Number and proportion of patients successfully referred by CHWs to BHCs.
- Proportion of malaria negative and positive given antibiotics at CHW and/or clinic level.

### Evaluation Criteria:

The evaluation criteria will follow that of the draft Community Based Management of Malaria guidelines. These represent national targets for inputs, outputs and outcome indicators with targets set by the National Programme and its partners.

Key indicators for the evaluation are laid out in the draft CMM strategy. At the time of writing this is still in draft and so these indicators and the outcome measures may change.

The key indicators and targets to monitor inputs, process and outcome of CBMM programme (taken from the Draft CMM Strategy for Afghanistan) are shown in annex 3.

## Outcome Measurement and Recording:

### Outcome Measurement:

In the community healthcare setting, there are several possible outcomes to any consultation with a CHW. Once the initial consultation is made, the CHW will decide on a diagnosis and then a specific course of action. In addition, patients may present at the clinic, either after a consultation with a CHW, or without a consultation. In the former case, the patient may have been referred, or may have self referred. The patient may also attend the clinic without having a consultation with a CHW (figure 3).

**Figure 3: Schematic diagram showing the potential path of a patient through the health system, starting at community level,**

Data and outcomes will be recorded at both the clinic and community level. The outcomes will be measured against PCR based diagnosis of malaria (including Pf, Pv, *P ovale* and *P malariae*). Filter paper blood spots will be collected at the diagnosis stage (at community and clinic level).

### Data recording:

Patient data will be recorded on pro-forma Patient Tracking Forms designed for each level of the study health system (i.e. CHW and clinic level).

The CHW record form is designed for semi-literate health-workers and uses both pictorial and textual components. All health workers will be trained by study staff (registrars) in filling out the form. The form will have been pretested and validated prior to commencement of the trial. The form will record the patients age and sex, symptoms (fever, cough, diarrhoea, shivering/rigors), length of time since symptoms began, diagnosis made (including whether an RDT was used and what the result was), and then the action taken, as in Figure 2. See annex 4).

For clinics, the form employed in the randomised trial has been adapted for use (see annex 5). The clinic physician (whether a doctor or not) will record the information at the time of the consultation.

Whether referred by CHWs or not, patients will be given an identifier card to present to the clinic to indicate that they were enrolled in the study. The referral note will have the same patient number as the enrolment data, which will be used to match the patient at the clinic with the same patient enrolled by the CHW.

## Sample Size and Statistical Methods:

### Sample Size Assumptions:

Key parameters and assumptions used to calculate the sample size are listed below:

- Number of clusters (clinics): 22
- Number of Health Posts: 207
- Number of CHWs: 414
- Expected difference in primary outcome in Phase 1 (40%):
  - Arm 1 - Non-intervention: 10% correctly treated vs.*
  - Arm 2 – RDT: 50% correctly treated*
- Expected difference in major secondary outcome (in phase 1):
  - Proportion of Pf positive patients receiving ACT: 0.5% in arm 1 vs. 50% in arm 2.*
  - Proportion of malaria negative patients treated with antimalarial: 90% in arm 1 vs. 50% in arm 2*
  - Proportion of malaria negative patients treated with antibiotic: 5% in non-intervention arm, 15% in RDT arm.
- Expected difference in primary outcome in Phase 2 (20%):
  - Arm 2 – RDT: 50% correctly treated.
  - Arm 3 – RDT extended programme: 70% or greater are correctly treated.
- Expected differences in major secondary outcome (in phase 2):
  - Proportion of Pf positive patients receiving ACT: 50% in arm 2 vs. 85% in arm 3.
  - Proportion of malaria negative patients treated with anti-malarial: 50% in RDT arm, 25% in RDT extended programme arm.
  - Proportion of malaria negative patients treated with antibiotic: 15 % in RDT arm, 20% in RDT extended arm.
- Sub-group analysis assumes that 40% of patients are under 10yrs*.

* estimates are based on initial results from the current randomised trial, based on data from 22 clinics in Afghanistan. There are no estimates in this region for effectiveness at CHW level.

### Number of consultations per clinic and health post:

In the 22 study clinic, there are 207 health posts (414 community health workers). The sample size will be limited by the number of consultations where an RDT will be deployed. The table shows the monthly activities report for the Health Posts in the study clinics for 3 months, from July-Sept 2010.

| **Province/clinic** | **Number of Health Posts** | **Number of treatments given for:** | | | |
| --- | --- | --- | --- | --- | --- |
|  |  | **Malaria** | **URTI** | **Diarrhoea** | **Total** |
| **Kunduz** | **65** | **47** | **2693** | **3161** | **5901** |
| Ajighan | 6 | 3 | 267 | 345 | 615 |
| AngorBagh | 7 | 3 | 568 | 823 | 1394 |
| ArbabRamazani | 2 | 2 | 54 | 73 | 129 |
| BolaQachi | 5 | 10 | 246 | 268 | 524 |
| CharSari | 6 | 2 | 245 | 205 | 452 |
| Khan Abad | 20 | 0 | 560 | 584 | 1144 |
| KhwajaGhaltan | 8 | 6 | 264 | 387 | 657 |
| Madrasa | 3 | 17 | 131 | 103 | 251 |
| Nick Pay | 3 | 0 | 36 | 49 | 85 |
| SandoqSai | 5 | 4 | 322 | 324 | 650 |
|  |  |  |  |  |  |
| **Nangarhar** | **142** | **2471** | **3736** | **3611** | **9818** |
| Baro | 8 | 63 | 158 | 230 | 451 |
| Batikot | 21 | 450 | 541 | 468 | 1459 |
| Behsood | 16 | 216 | 354 | 297 | 867 |
| Chardhi | 6 | 45 | 112 | 93 | 250 |
| GardiGhos | 6 | 310 | 342 | 281 | 933 |
| Khewa | 12 | 158 | 487 | 670 | 1315 |
| LandaBuch | 10 | 174 | 258 | 247 | 679 |
| MamandDara | 11 | 382 | 319 | 313 | 1014 |
| NajmulQura | 6 | 189 | 291 | 329 | 809 |
| Rodat | 13 | 52 | 50 | 37 | 139 |
| SangarSarai | 18 | 212 | 271 | 338 | 821 |
| Shegay | 15 | 220 | 553 | 308 | 1081 |
| **Grand Total** | **207** | **2518** | **6429** | **6772** | **15719** |

### Sample size:

An initial sample size of 1500 was selected for phase I to assess the secondary outcome of number of malaria positive patients correctly treated. Because Pf malaria is rare, the study is not powered for assessing the proportion of Pf cases correctly treated, although this will be examined as an exploratory analysis. For Phase II an initial sample size of 4000 individuals would assess the same secondary outcome, adjusted for cluster sampling. The confidence level (alpha) is 0.05 and the power (beta) is 0.8.

The sample size for both phases is adjusted for both intra-cluster variation (intra-cluster correlation coefficient of 0.5). To give a harmonic mean (see section 2.5.3) of 68 patients per cluster for phase I and 182 for phase II sample sizes of 2542 and 6792 are required in phase I and phase II respectively. This step is discussed in more detail in section 2.5.3.

|  |  | **Phase I** | | **Phase II** | |
| --- | --- | --- | --- | --- | --- |
| **Clinic** | **Province** | **SS weighting by cluster size** | **SS Weighted by cluster size for harmonic mean of 68** | **SS weighting by cluster size** | **SS Weighted by cluster size for harmonic mean of 182** |
| Ajighan | KDZ | 73 | 124 | 194 | 330 |
| AngorBagh | KDZ | 163 | 276 | 435 | 739 |
| ArbabRamazani | KDZ | 15 | 25 | 41 | 70 |
| BolaQachi | KDZ | 66 | 112 | 175 | 297 |
| CharSari | KDZ | 52 | 88 | 138 | 234 |
| Khan Abad | KDZ | 147 | 249 | 392 | 666 |
| KhwajaGhaltan | KDZ | 66 | 112 | 176 | 299 |
| Madrasa | KDZ | 30 | 51 | 80 | 136 |
| Nick Pay | KDZ | 12 | 20 | 33 | 56 |
| SandoqSai | KDZ | 63 | 107 | 169 | 287 |
| Baro | NGR | 37 | 63 | 100 | 170 |
| Batikot | NGR | 121 | 205 | 322 | 547 |
| Behsood | NGR | 72 | 122 | 191 | 324 |
| Chardhi | NGR | 21 | 36 | 55 | 93 |
| GardiGhos | NGR | 77 | 130 | 206 | 350 |
| Khewa | NGR | 109 | 185 | 290 | 493 |
| LandaBuch | NGR | 56 | 95 | 150 | 255 |
| MamandDara | NGR | 84 | 142 | 224 | 380 |
| NajmulQura | NGR | 67 | 114 | 178 | 302 |
| Rodat | NGR | 12 | 20 | 31 | 53 |
| SangarSarai | NGR | 68 | 115 | 181 | 307 |
| Shegay | NGR | 89 | 151 | 238 | 404 |
| Sample Size | | 1500 | 2542 | 3999 | 6792 |
|  |  |  |  |  |  |
| Arithmetic Mean | | 68.1818182 | 115.5455 | 181.7727273 | 308.7273 |
| Harmonic Mean | | 40.1240439 | 67.61738 | 107.1515805 | 182.1944 |
| Ratio (arithmetic:harmonic) | | 1.69474 |  | 1.69853 |  |

### Statistical Methods:

An analysis plan will be developed by the investigators uring the trial. This will be reviewed by the statistician and DSMB prior to use. Broadly, primary and secondary outcomes will be assessed for differences by intervention arm using proportions with appropriate measures of statistical significance, for example chi^2^ test or Fisher’s exact test. Multivariate analysis will be conducted using a variety of potential explanatory variables related to the prescriber and the patients. These parameters are:

- Prescriber variables: Training history (prior to the intervention), age, gender.
- Patient variables: Age, gender, severity of symptoms, range of symptoms.
- Health system variables: clinic type, place of diagnosis and treatment (CHW or clinic).

## Laboratory Methods:

### RDT Selection:

In-line with national policy the study will use: CareStart™ 3-line Pf (HRP2) + Pan (pLDH) malaria rapid diagnostic test (AccessBio, New Jersey, USA). Recently, this test was evaluated in Afghanistan and found to have accuracy >95% and to match the WHO criteria for accuracy.

### Sample Collection:

Filter paper blood spots will be collected from participants. These will be collected using clean disposable lancets and gloves at the same time as the RDT is used. If the RDT is not used (i.e. in the non-intervention group) then the filter paper will be collected during the consultation. Filter papers will be stored in ziplock bags with silica beads. They will be returned to the clinic within 7 days and stored in a refrigerator (4^o^C) until shipped to Kabul for evaluation (see 2.8.4, below)

### RDT Accuracy:

RDT accuracy and, importantly, user interpretation of the results will be assessed under field conditions.

RDT accuracy will be determined by comparing PCR results for filter paper blood spots as the gold standard against the RDT result noted on the case-record form.

The accuracy of user interpretation will be determined by cross-checking RDT results noted by CHWs on each case record form, with RDT results recorded separately by an independent evaluator (i.e. all RDTs will be double-blind read). Because RDTs are liable to become falsely positive if exposed to humidity post-execution, CHWs will be instructed to store all RDTs in zip-lock bags with silica gel immediately after reading. The stored RDTs will be collected by field workers and undergo a second reading/interpretation by study registrars within 7 days.

### PCR Evaluation:

Filter paper blood spots will be collected in triplicate for all patients enrolled in the study and processed in Kabul. As described previously, DNA will be extracted with the crude TE buffer method[1], while the Mangold melt-curve real-time PCR[2] will be run to detect *Plasmodium* sp. DNA and discriminate between species. The assay will not be performed quantitatively. A random selection of 10% of all filter paper blood spots will be sent to the LSHTM malaria reference centre for re-evaluation by alternative PCR methods and quality control.

## Ethical Issues:

### Informed consent:

Written informed consent will be obtained from all study participants, including the community health workers. A consent form will be applied to the CHWs prior to the start of the study and they will be free to object to their inclusion in the research (annex 8).

Patients consulting with CHWs will be asked to provide informed consent following a verbal description of the risks, benefits and procedures and will be asked to sign or mark the form to indicate that they agree to take part in the study.

### Confidentiality:

Patient identities will only be known to the CHWs. The CHWs will keep a record of who the patients are, but this is only to be accessed in the case of mistreated Pf malaria (see below). On forms used for noting routine data, only the patient number will be noted, so no personal identifying information will be available to anyone.

### Risks and potential adverse events:

There are minimal risks associated with this study. Blood samples would, in most cases, be taken anyway for the application of the RDT is associated with minimal risk of secondary infection at the puncture site and with mild pain.

The main risk is in the mistreatment of falciparum malaria with either no antimalarial or with an ineffective antimalarial (i.e. CQ). Currently, falciparum malaria is quite rare in the study areas (although it may vary seasonally and geographically) so the risk of this occurrence is low. According to the National Guidelines and the study protocol, all Pf positive patients will be referred to a clinic for further evaluation which further reduces the risk of this occurring, since all clinics will have parasitological diagnosis available (either through RDT or through microscopy).

The trial intervention arm is unlikely to increase this risk, in fact, rather the opposite. This potential adverse event will be monitored throughout the trial and its occurrence will be reported to the Data Safety and Monitoring Board (DSMB). Immediately following a PCR result which is positive for Pf (regardless of coinfection), results from the patient record form will be cross checked to see which treatment was given. Because of logistical constraints, PCR results will not be known for more than 2 weeks after the initial assessment of the patient. Every effort will be made to trace the patient and ensure that they have recovered. If not, they will be referred to the health facility for further treatment.

Depending on the frequency with which this event occurs, the study staff will inform the DSMB of any action being taken which could include re-training of staff or withdrawal of CHWs from the trial. All efforts will be made to follow-up the patient within a few days of knowing the PCR result. All patients will be advised by CHWs to seek treatment at the clinic if symptoms recur or continue following the treatment.

# Programme Implementation:

## Staff structure:

The staff structure will match the current setup, although roles will be slightly changed. The staff structure is as below:

## Staff roles:

- - 1. Technical Team: The technical teams consists of the Principle Investigators, Data Coordinator, the HN-TPO Deputy Programme Manager, and Merlin’s Country Health Director. This team is responsible for all management issues related to the project.
    2. Provincial management teams are responsible for implementing the programme according to the protocol, ensuring that data is adequately recorded and entered and ensuring that all staff have adequate training. They are responsible for monitoring of progress and reporting of adverse events.
    3. Field Workers: Are each responsible for a cluster of 3-4 clinics. They are responsible for supervising the registrars and ensuring that the project is conducted per protocol, reporting of adverse events, and ensuring that data and samples are stored and transported appropriately.
    4. Registrars: The registrars (one per clinic) will each supervise between 3 and 10 CHWs and the CHS of the clinic. This will be to ensure that they are collecting informed consent from patients, recording data correctly, bringing samples and patient record to the clinic on a regular basis (once per week). Registrars are also responsible for handling referred cases and ensuring that their data is noted atthe clinic level.
    5. CHWs – Will be participants in the study, in that their decisions in diagnosis will be recorded. They will also be collecting data on the patient tracking form.

## Staff training:

Training of all provincial and clinic staff will be conducted by senior technical staff and project managers in the provinces. Registrars will be trained in the study protocol and SOPs, including ethical issues, blood safety, data recording, data processing and study monitoring.

CHWs will be trained according to the intervention arm, but all will be trained to the same standard in blood safety, enrolment characteristics, the consent process and reporting of adverse events.

**Curriculum for CHS and CHW training:**

| **Training Topic** | **Contents** | **Registrars** | **Arm 1** | **Arm 2** | **Arm 3** |
| --- | --- | --- | --- | --- | --- |
|  |  |  |  |  |  |
| *Trial Procedures* | 2 day | Trainer: Conducted by ACTc senior researchers | | | |
| Introductory training | Objectives and overview of the study – tone is not on treatment accuracy, but on different methods for diagnosis and treatment of illness at community level |  | **√** | **√** | **√** |
| Form Filling | Introduction to the form – what information is collected  Each section of the form  How to fill the form (ticking boxes)  Making corrections of mistakes  Practice using a series of case studies | **√** | **√** | **√** | **√** |
| Specimen Collection | Blood and sharps safety  Taking a blood spot  Storing the blood spot in package | **√** | **√** | **√** | **√** |
| Package training / equipment provided | Package contents  Patient numbers  Process for enrolling patients – consent forms  Handing the package to the registrar | **√** | **√** | **√** | **√** |
| Ethics and Consent | Basic Ethics – human use in research.  Consent – what is it? How to get it? | **√** | **√** | **√** | **√** |
| Fraud and bogus data collection | What happens?  How do we detect it? | **√** | **√** | **√** | **√** |
| Basic SoP for field data collection | Run through the CHW SoP for recruitment | **√** | **√** | **√** | **√** |
| Advanced Training in trial SoP | Covers all sections from Registrar and below | **√** |  |  |  |
| *Intervention Training* |  |  |  |  |  |
| *Length and Trainers* | 2 days for clinics in intervention arms | Trainers: Master trainers – from NGOs and MRC staff; Dr Abid (HealthNet malaria master trainer, JAA) and Dr Faiz (HealthNet malaria master trainer, KDZ). Master trainers will train CHSs who will train CHWs in the intervention arm 1. | | | |
| Refresher Training of ToT |  | For Master Trainers in Arm 1 | | | |
| RDT Training* | Copied from the CBMM and any other training documents developed by NMLCP and partners |  | **√ -** possibly without RDT | **√** | - |
| RDT+ Training | Identified by ACTc team and added for phase II |  | - |  | **√** |

* Master trainers trained by NMLCP; Master trainers CHWs (in their own clinics).

## Implementation Plan:

The initial plan for implementation is described in the table below:

|  | April | | | | May | | | | June | | | | July | | | | Aug | | | | Sept | | | | Oct | | | | Nov | | | | Dec | | | |
| --- | --- | --- | --- | --- | --- | --- | --- | --- | --- | --- | --- | --- | --- | --- | --- | --- | --- | --- | --- | --- | --- | --- | --- | --- | --- | --- | --- | --- | --- | --- | --- | --- | --- | --- | --- | --- |
|  | 1 | 2 | 3 | 4 | 1 | 2 | 3 | 4 | 1 | 2 | 3 | 4 | 1 | 2 | 3 | 4 | 1 | 2 | 3 | 4 | 1 | 2 | 3 | 4 | 1 | 2 | 3 | 4 | 1 | 2 | 3 | 4 | 1 | 2 | 3 | 4 |
| **Activity** |  |  |  |  |  |  |  |  |  |  |  |  |  |  |  |  |  |  |  |  |  |  |  |  |  |  |  |  |  |  |  |  |  |  |  |  |
|  |  |  |  |  |  |  |  |  |  |  |  |  |  |  |  |  |  |  |  |  |  |  |  |  |  |  |  |  |  |  |  |  |  |  |  |  |
| **Study Preparation:** |  |  |  |  |  |  |  |  |  |  |  |  |  |  |  |  |  |  |  |  |  |  |  |  |  |  |  |  |  |  |  |  |  |  |  |  |
| Ethics Review complete (London and Kabul) |  |  |  |  |  |  |  |  |  |  |  |  |  |  |  |  |  |  |  |  |  |  |  |  |  |  |  |  |  |  |  |  |  |  |  |  |
| Purchasing of key items |  |  |  |  |  |  |  |  |  |  |  |  |  |  |  |  |  |  |  |  |  |  |  |  |  |  |  |  |  |  |  |  |  |  |  |  |
| Training of Technical Staff |  |  |  |  |  |  |  |  |  |  |  |  |  |  |  |  |  |  |  |  |  |  |  |  |  |  |  |  |  |  |  |  |  |  |  |  |
| Training of Field Staff |  |  |  |  |  |  |  |  |  |  |  |  |  |  |  |  |  |  |  |  |  |  |  |  |  |  |  |  |  |  |  |  |  |  |  |  |
| Pre-intervention Survey |  |  |  |  |  |  |  |  |  |  |  |  |  |  |  |  |  |  |  |  |  |  |  |  |  |  |  |  |  |  |  |  |  |  |  |  |
| Training of CHWs |  |  |  |  |  |  |  |  |  |  |  |  |  |  |  |  |  |  |  |  |  |  |  |  |  |  |  |  |  |  |  |  |  |  |  |  |
|  |  |  |  |  |  |  |  |  |  |  |  |  |  |  |  |  |  |  |  |  |  |  |  |  |  |  |  |  |  |  |  |  |  |  |  |  |
| **Study Implementation** |  |  |  |  |  |  |  |  |  |  |  |  |  |  |  |  |  |  |  |  |  |  |  |  |  |  |  |  |  |  |  |  |  |  |  |  |
| Enrolment of Patients (Phase 1) |  |  |  |  |  |  |  |  |  |  |  |  |  |  |  |  |  |  |  |  |  |  |  |  |  |  |  |  |  |  |  |  |  |  |  |  |
| Enrolment of Patients (Phase 2) |  |  |  |  |  |  |  |  |  |  |  |  |  |  |  |  |  |  |  |  |  |  |  |  |  |  |  |  |  |  |  |  |  |  |  |  |
| Laboratory work (PCR) |  |  |  |  |  |  |  |  |  |  |  |  |  |  |  |  |  |  |  |  |  |  |  |  |  |  |  |  |  |  |  |  |  |  |  |  |
| Laboratory work (Reference microscopy) |  |  |  |  |  |  |  |  |  |  |  |  |  |  |  |  |  |  |  |  |  |  |  |  |  |  |  |  |  |  |  |  |  |  |  |  |
|  |  |  |  |  |  |  |  |  |  |  |  |  |  |  |  |  |  |  |  |  |  |  |  |  |  |  |  |  |  |  |  |  |  |  |  |  |
| **Data Analysis and Reporting** |  |  |  |  |  |  |  |  |  |  |  |  |  |  |  |  |  |  |  |  |  |  |  |  |  |  |  |  |  |  |  |  |  |  |  |  |
| Completion of data entry |  |  |  |  |  |  |  |  |  |  |  |  |  |  |  |  |  |  |  |  |  |  |  |  |  |  |  |  |  |  |  |  |  |  |  |  |
| Interim Data analysis and Evaluation |  |  |  |  |  |  |  |  |  |  |  |  |  |  |  |  |  |  |  |  |  |  |  |  |  |  |  |  |  |  |  |  |  |  |  |  |

## References

Bereczky S, Martensson A, Gil JP, Farnert A: **Short report: rapid DNA extraction from archive blood spots on filter paper for genotyping of Plasmodium falciparum**. *The American journal of tropical medicine and hygiene* 2005, **72**(3):249-251.

Mangold KA, Manson RU, Koay ESC, Stephens L, Regner MA, Thomson Jr RB, Peterson LR, Kaul KL: **Real-time PCR for detection and identification of *Plasmodium* spp**. *Journal of clinical microbiology* 2005, **43**(5):2435-2440.

Raab, G.M and Butcher, I. Balance in cluster randomized trials. Statistics in Medicine 2001; 20: 351-65.

Moulton, L.H. Covariate-based constrained randomization of group-randomized trials. Clinical Trials 2004; 1: 297-305.

Sismanidis, C. et al. Restricted randomisation of ZAMSTAR: a 2 x 2 factorial cluster randomized trial. Clinical Trials 2008; 5: 316-327

Nietert, P.J. et al. An application of a modified constrained randomization process to a practice-based cluster randomized trial to improve colorectal cancer screening. Contemporary Clinical Trials, 2008; 30: 129-13

## Annex 1: CHW enrolment and activity form:

| **Biographic details** | | | | **Educational background** | | | | | | | | **ACTC CRT enrolment survey with CHWs**  *Instructions for use: Complete every question. Make sure that the answers are written clearly.* | |
| --- | --- | --- | --- | --- | --- | --- | --- | --- | --- | --- | --- | --- | --- |
| 1 | CHW ID number: CRT _ ClinicID_HP_CHW | [Insert study label] | | 6 | What level of schooling did the CHW complete? | | | No education | | | \|__\| |  |  |
|  |  |  |  |  |  |  |  | Informal / religious | | | \|__\| |  |  |
|  |  |  |  |  |  |  |  | Primary | | | \|__\| |  |  |
|  |  |  |  |  |  |  |  | Secondary | | | \|__\| |  |  |
|  |  |  |  |  |  |  |  | Higher | | | \|__\| |  |  |
| 2 | Health-Post MoPH No: | \|__\|__\|__\|__\|__\| | |  | | | | | | | |  |  |
| 3 | CHW Gender: | Female | \|__\| | 7 | Literacy level: | | | Illiterate | | | \|__\| |  |  |
|  |  | Male | \|__\| |  |  |  |  | Semi-literate | | | \|__\| |  |  |
| 4 | CHW age: | \|__\|__\|yrs | |  |  |  |  | Literate | | | \|__\| |  |  |
| 5 | Village CHW is based: |  | |  |  |  |  |  |  |  |  |  |  |
| **Job role** | | | | **CHW training** | | | | | | | |  |  |
| 8 | Length of time as CHW:  (Eg: 00yrs 09months) | \|__\|__\|yrs  \|__\|__\|mths | | 13 | Received initial training at beginning of CHW job? | Yes | | | | \|__\| | |  |  |
|  |  |  |  |  |  | No | | | | \|__\| | |  |  |
| 9 | Does CHW work with a partner? | Yes | \|__\| | 14 | Number of weeks spent on CHW initial training: | \|__\|__\| weeks | | | | | |  |  |
|  |  | No | \|__\| | 15 | Received any other CHW training whilst a CHW? | Yes | | | | \|__\| | |  |  |
|  |  |  |  |  |  | No | | | | \|__\| | |  |  |
| 10 | If yes to what is the partner’s ID number: | CRT-\|__\|__\|__\|-\|__\|__\|-\|__\| | | 16 | If yes to Q14, list the training received (for CHW role) and who provided the training: | Training Subject | | | | Training Provider | | No. Days | Year |
| 11 | Does CHW have another job as well as this job? | Yes | \|__\| |  |  |  | | | |  | | \|__\|__\| | \|__\|__\|__\|__\| |
|  |  | No | \|__\| |  |  |  | | | |  | | \|__\|__\| | \|__\|__\|__\|__\| |
| 12 | If yes to Q,10 write name of job |  | |  |  |  | | | |  | | \|__\|__\| | \|__\|__\|__\|__\| |
|  | | | |  |  |  | | | |  | | \|__\|__\| | \|__\|__\|__\|__\| |
|  |  |  |  |  |  |  | | | |  | | \|__\|__\| | \|__\|__\|__\|__\| |
| **Socioeconoimc status** | | | | | | | | | | | |  | |
| 17 | Household assets: (tick all that apply) | TV [ ] | | Radio [ ] | | | Car [ ] | | Pressure Cooker [ ] | | |  |  |
|  |  | Land [ ] | | House [ ] | | | Business [ ] | | Guest room [ ] | | |  |  |
| 18 | Amount of land owned: | \|__\|__\|jeribs | | 19. Number of people in household: | | | \|__\|__\| people | | | | |  |  |
| 20 | Number of rooms: | \|__\|__\| rooms | | 21. Is CHW the primary wage earner in their household? | | | Yes | | \|__\| | | |  |  |
|  |  |  |  |  |  |  | No | | \|__\| | | |  |  |

***Instructions for use****: The aim of this survey is to understand the kind of activities that a CHW carries out during a typical working week. The study registrar should explain the following to the CHW –*

- *This is not a test, we need to get an idea of what kind of things the CHWs do and how long they spend doing each activity.*
- *We are interested in what activities they can do during a typical week as well as what activities they are not able to do during a typical week, for instance if they do not do a particular activity, perhaps because they do not have enough time, this is useful information.*
- *The CHWs should be completely confident that this information is confidential and will not be shared with their employers.*

Top of Form

| **CHW details** | |
| --- | --- |
| 1. CHW ID number: CRT _ Clinic ID_ HP #_CHW # | CRT -\|__\|__\|__\| - \|__\|__\|-\|__\| |
| 1. Health-post MoPH number: | \|__\|__\|__\|__\|__\| |
| **CHW workload** | |
| 1. In a typical week, how many days of the week do you conduct CHW activities, for instance, every day or less than every day? (0-7 days) | \|__\|__\|days |
| 1. On a typical day when you work as a CHW, how many hours do you spend on CHW activities? | \|__\|__\| hrs \|__\|__\|mins |

| **A) Has the CHW ever conducted the following activity as part of their role?** | | **B) If yes, in the previous week, how many times did the CHW carry out this activity?** | **C) When CHW last carried out this activity / consultation – how many hours (or minutes) did the activity take?** | **D) When the CHW last carried out this activity - where did it take place?** | |
| --- | --- | --- | --- | --- | --- |
| 1. Consultation for pregnancy | Yes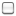 No 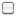 | \|__\|__\| times | \|__\|__\| hrs \|__\|__\|mins | CHW’s home | 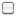 |
|  |  |  |  | Community centre | 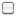 |
|  |  |  |  | Nearest clinic | 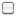 |
|  |  |  |  | Patient’s home | 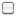 |
|  |  |  |  | Outside in open | 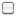 |
|  |  |  |  | Other | 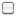 |
| 1. Consultation for a sick adult (excluding pregnant mothers) | Yes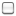 No 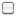 | \|__\|__\| times | \|__\|__\| hrs \|__\|__\|mins | CHW’s home | 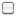 |
|  |  |  |  | Community centre | 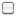 |
|  |  |  |  | Nearest clinic | 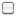 |
|  |  |  |  | Patient’s home | 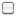 |
|  |  |  |  | Outside in open | 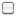 |
|  |  |  |  | Other | 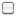 |
| 1. Consultation for a sick child (excluding pregnant mothers) | Yes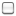 No 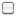 | \|__\|__\| times | \|__\|__\| hrs \|__\|__\|mins | CHW’s home | 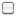 |
|  |  |  |  | Community centre | 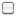 |
|  |  |  |  | Nearest clinic | 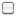 |
|  |  |  |  | Patient’s home | 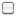 |
|  |  |  |  | Outside in open | 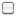 |
|  |  |  |  | Other | 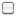 |
| 1. Treatment for accident / trauma / other injury | Yes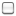 No 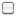 | \|__\|__\| times | \|__\|__\| hrs \|__\|__\|mins | CHW’s home | 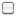 |
|  |  |  |  | Community centre | 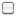 |
|  |  |  |  | Nearest clinic | 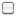 |
|  |  |  |  | Patient’s home | 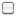 |
|  |  |  |  | Outside in open | 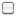 |
|  |  |  |  | Other | 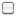 |
| 1. Vaccine program activity | Yes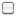 No 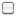 | \|__\|__\| times | \|__\|__\| hrs \|__\|__\|mins | CHW’s home | 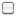 |
|  |  |  |  | Community centre | 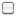 |
|  |  |  |  | Nearest clinic | 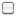 |
|  |  |  |  | Patient’s home | 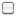 |
|  |  |  |  | Outside in open | 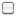 |
|  |  |  |  | Other | 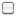 |
| 1. Family planning | Yes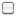 No 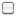 | \|__\|__\| times | \|__\|__\| hrs \|__\|__\|mins | CHW’s home | 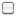 |
|  |  |  |  | Community centre | 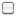 |
|  |  |  |  | Nearest clinic | 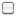 |
|  |  |  |  | Patient’s home | 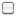 |
|  |  |  |  | Outside in open | 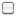 |
|  |  |  |  | Other | 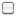 |
| 1. Delivering bed nets to houses | Yes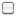 No 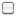 | \|__\|__\| times | \|__\|__\| hrs \|__\|__\|mins | CHW’s home | 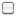 |
|  |  |  |  | Community centre | 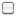 |
|  |  |  |  | Nearest clinic | 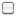 |
|  |  |  |  | Patient’s home | 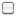 |
|  |  |  |  | Outside in open | 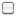 |
|  |  |  |  | Other | 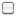 |
| 1. Taking part in health committee meetings | Yes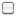 No 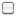 | \|__\|__\| times | \|__\|__\| hrs \|__\|__\|mins | CHW’s home | 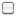 |
|  |  |  |  | Community centre | 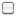 |
|  |  |  |  | Nearest clinic | 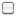 |
|  |  |  |  | Patient’s home | 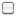 |
|  |  |  |  | Outside in open | 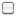 |
|  |  |  |  | Other | 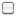 |
| 1. Filling in MAR reports | Yes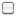 No 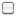 | \|__\|__\| times | \|__\|__\| hrs \|__\|__\|mins | CHW’s home | 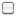 |
|  |  |  |  | Community centre | 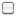 |
|  |  |  |  | Nearest clinic | 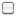 |
|  |  |  |  | Patient’s home | 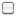 |
|  |  |  |  | Outside in open | 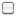 |
|  |  |  |  | Other | 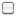 |
| 1. Health education and awareness | Yes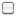 No 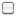 | \|__\|__\| times | \|__\|__\| hrs \|__\|__\|mins | CHW’s home | 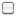 |
|  |  |  |  | Community centre | 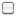 |
|  |  |  |  | Nearest clinic | 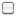 |
|  |  |  |  | Patient’s home | 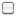 |
|  |  |  |  | Outside in open | 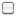 |
|  |  |  |  | Other | 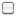 |
| 1. Attending training sessions | Yes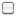 No 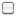 | \|__\|__\| times | \|__\|__\| hrs \|__\|__\|mins | CHW’s home | 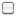 |
|  |  |  |  | Community centre | 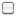 |
|  |  |  |  | Nearest clinic | 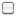 |
|  |  |  |  | Patient’s home | 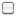 |
|  |  |  |  | Outside in open | 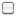 |
|  |  |  |  | Other | 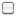 |

| **CHW travel to the health clinic** | |
| --- | --- |
| 1. How long does it take you to get to your clinic? | \|__\|__\| hrs \|__\|__\| mins |
| 1. What transport do you normally use to get to the clinic? | __________________________ |
| 1. Last week, how many times did you go to the clinic? | \|__\|__\| times |

Bottom of Form

Top of Form

Bottom of Form

## Annex 2: Revised IMCI algorithm for treatment of fever at community level:

Source: DRAFT Strategy for Community Based Management of Malaria, WHO/MoPH, 2011.


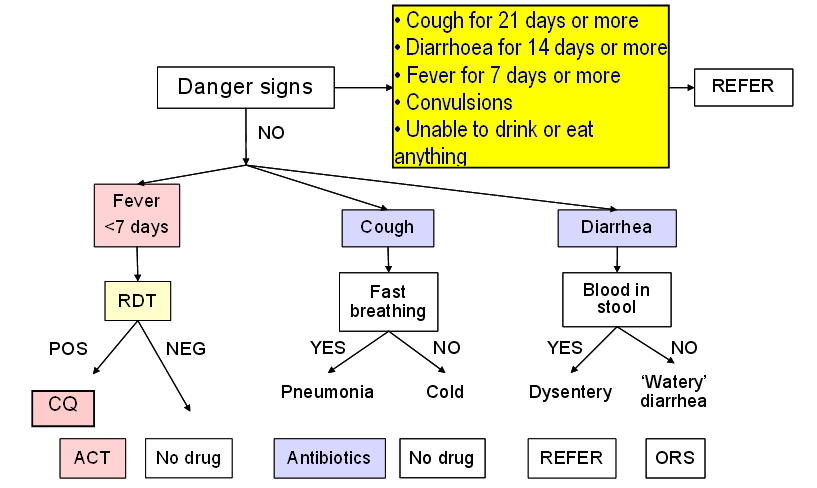


Algorithm for managing common childhood illness at community level as part of the Community IMCI strategy^[[4]](#footnote-4)^.

## Annex 3: Evaluation indicators for National CBMM strategy (benchmarks for Arm 2).

| # | **Indicator** | **Data Requirement** | **Notes (added by the Investigators)** |
| --- | --- | --- | --- |
| 1 | Number of RDT received in the country by implementing agencies | Number of RDTs received in the country per year by funding and implementing agencies | Not for use in the evaluation |
| 2 | Number of RDT delivered to BPHS implementers at provincial level | Number of RDTs delivered at provincial level  to BPHS implementers | Not for use in the evaluation |
| 3 | Proportion of targeted BHCs and HSCs reporting no RDT stockouts | **Numerator:** Number of targeted BHCs and HSCs reporting no RDT stockouts per month **Denominator:** Number of targeted BHCs and HSCs submitting monthly stock reports on RDT | Not for use in the evaluation – the trial will include the assumption that RDTs are available in the clinics in abundance and they will be provided with sufficient tests to reach the sample size requirements |
| 4 | Proportion of targeted HPs reporting no RDT stockouts | **Numerator:** Number of targeted HPs reporting no RDT stockouts on tally sheets  **Denominator:** Number of targeted HPs submitting tally sheets with RDT stocks | As above |
| 5 | Number of ACT received in the country by implementing agencies | Number of ACTs received in the country per year by funding and implementing agencies | Not used in the evaluation |
| 6 | Number of ACT delivered to BPHS implementers at provincial level | Number of ACTs delivered at provincial level  to BPHS implementers (every 6 months) | Not used in the evaluation |
| 7 | Proportion of targeted BHCs and HSCs reporting no ACT stockouts | **Numerator:** Number of targeted BHCs and HSCs reporting no ACT stockouts per month **Denominator:** Number of targeted BHCs and HSCs submitting monthly stock reports on ACT | Not used in the evaluation |
| 8 | Proportion of targeted HPs reporting no ACT stockouts | **Numerator:** Number of targeted HPs reporting no ACT stockouts on tally sheets  **Denominator:** Number of targeted HPs submitting tally sheets with ACT stocks | Not used in the evaluation |
| 9 | Proportion of malaria cases confirmed by RDT in targeted BHCs and HSCs | **Numerator:** Number of reported malaria cases confirmed by RDT in targeted BHCs and HSCs **Denominator:** All reported malaria cases from targeted BHCs and HSCs | Not used in the evaluation |
| 10 | Proportion of malaria cases confirmed by RDT in targeted Health Posts | **Numerator:** Number of reported malaria cases confirmed by RDT in targeted BHCs, HSCs and Health Posts  **Denominator:** All reported malaria cases from targeted BHCs, HSCs and Health Posts | Included in the evaluation.  The denominators will be (a) the number of suspected malaria cases (fever) and (b) the number of PCR confirmed malaria cases.  Draft Target: 85% |
| 11 | Proportion of reported falciparum cases confirmed by RDT treated with ACTs in targeted BHCs and HSCs | **Numerator:** Number of reported falciparum cases confirmed by RDT treated with ACTs in targeted BHCs and HSCs  **Denominator:** All reported falciparum cases confirmed by RDT from targeted BHCs and HSCs | Included in the evaluation.  The denominators will be (a) the number of suspected malaria cases (fever) and (b) the number of PCR confirmed malaria cases.  Draft Target (taken from national malaria strategic plan): 90% |
| 12 | Proportion of reported falciparum cases confirmed by RDT treated with ACTs in targeted Health Posts | **Numerator:** Number of reported falciparum cases confirmed by RDT treated with ACTs in targeted BHCs, HSCs and Health Posts  **Denominator:** All reported falciparum cases confirmed by RDT from targeted BHCs, HSCs and Health Posts | Included in the evaluation.  The denominators will be (a) the number of suspected malaria cases (fever) and (b) the number of PCR confirmed malaria cases.  Draft Target (taken from national malaria strategic plan): 90% |
| 13 | Proportion of reported non-falciparum cases confirmed by RDT treated with chloroquine + primaquine in targeted BHCs and HSCs | **Numerator:** Number of reportd non-falciparum cases confirmed by RDT treated with chloroquine + primaquine in targeted BHCs and HSCs  **Denominator:** All reported non-falciparum cases confirmed by RDT from targeted BHCs and HSCs | Included in the evaluation.  The denominators will be (a) the number of suspected malaria cases (fever) and (b) the number of PCR confirmed malaria cases.  Draft Target (taken from national malaria strategic plan): 90% |
| 14 | Proportion of reported non-falciparum cases confirmed by RDT treated with chloroquine in targeted Health Posts | **Numerator:** Number of reportd non-falciparum cases confirmed by RDT treated with chloroquine in targeted Health Posts  **Denominator:** All reported non-falciparum cases confirmed by RDT from targeted HPs | Included in the evaluation.  The denominators will be (a) the number of suspected malaria cases (fever) and (b) the number of PCR confirmed malaria cases.  Draft Target (taken from national malaria strategic plan): 90% |

## Annex 4: CHW Data collection form – this page is the front, and next page is the reverse of the form.


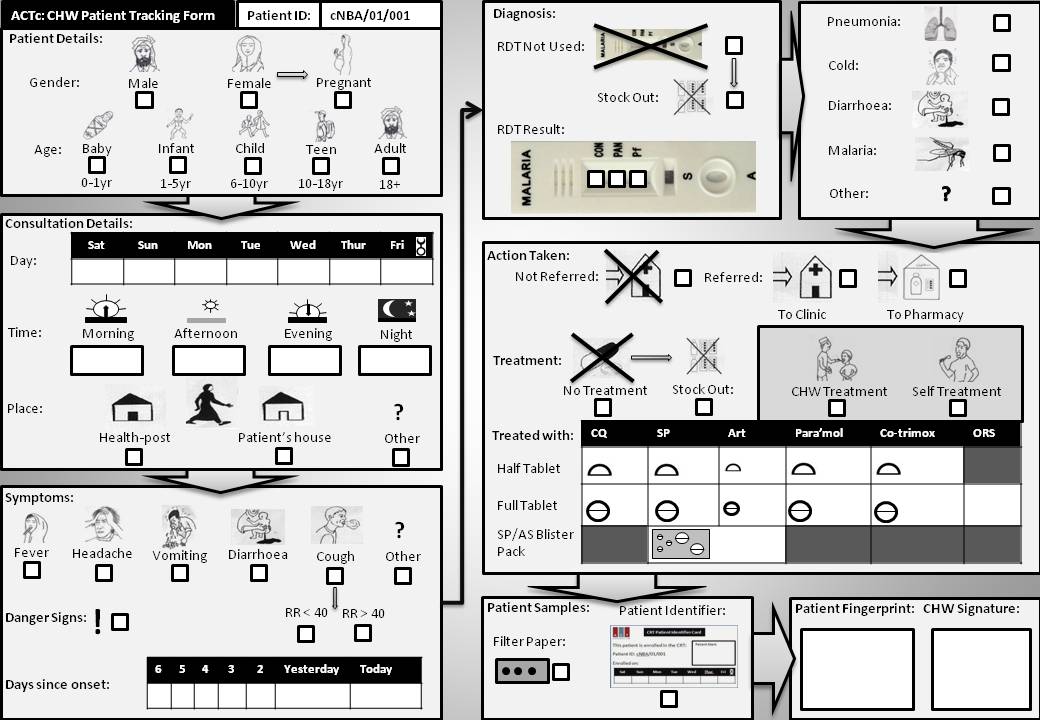


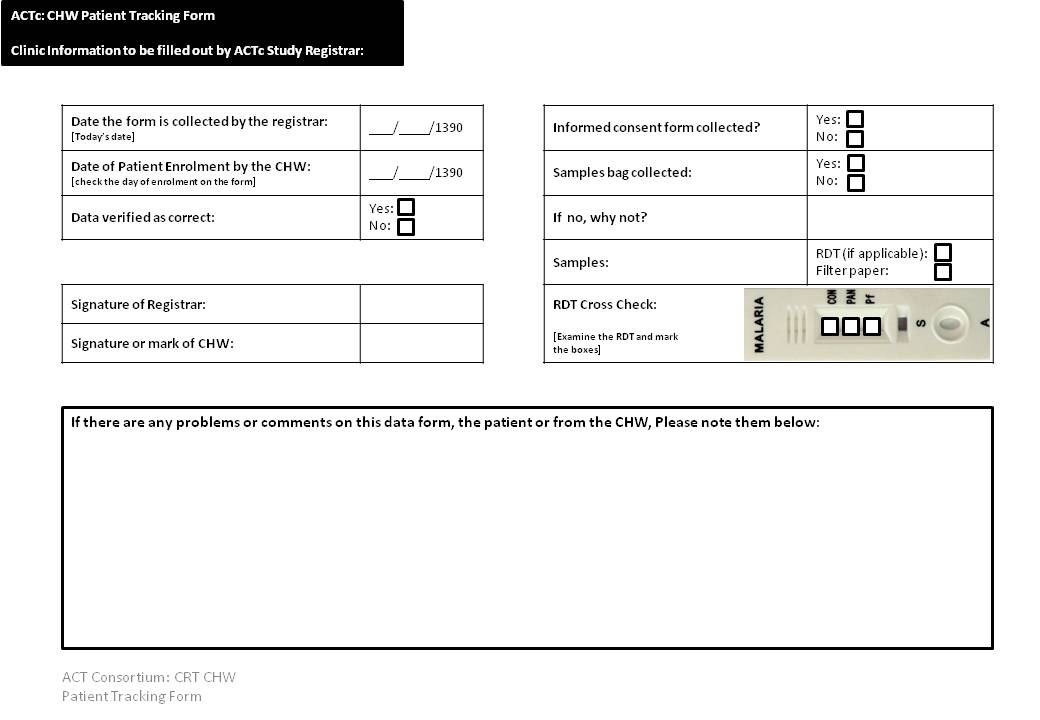


## Annex 5: Clinic Patient Tracking Form:

**A. Patient details:** (Study registrar – please fill out this section)

(Attach the patient ID sticker)

**1. Patient ID number:**

**2. CRT Patient ID Card Number: None (not enrolled)**  **Yes**  **-** **CRT ID:**

**3. Was the Patient Referred: No**  **Yes, referred by CHW**  **by other clinic**

**by family member**

**by other**

**4. Did the patient consult a CHW before attending the clinic:**

**No**  **Yes**  *- Which CHW (write name and village):*

CHW Name: Village:

**5. Patient name:**

**6. Sex: Male**  **Female**

**7. Age:** [  ][  ] **years**

**8. Date of Visit:** [  ][  ] - [  ][  ] - **1390** (Persian format: DD-MM-YYYY)

**9. Reference sample: Blood spot**  **(please tick √ when collected)**

**B. Clinician details:** (Clinician – please write your name & mark your job title/position in the box below)

**1. Name of clinician:**

**2. Position of clinician: MD (**♂**)**  **MD (**♀**)**  **Nurse**  **CHS**  **Midwife**

**C. Patient history & clinical exam:** (Clinician - please write patient signs and symptoms in this box)

**1. _______________________________________________________________________**

**2. _______________________________________________________________________**

**D. Clinic Diagnosis result:** (Clinic microscopist – please record the slide results in this box)

**No Laboratory Diagnosis:**

**Clinic slide result: *P. falciparum***  ***P. vivax***  **Mixed**  **Negative**

**RDT result:**

**F. Clinician’s diagnosis:** (Clinician - please write all the final diagnoses for the patient in this box)

**1. _______________________________________________________________________**

**2. _______________________________________________________________________**

**G. Prescription:** (Clinician – please write the patient’s full prescription in the box below)

**Anti-malarial**  **Antipyretics**  **Antibiotics**  **Other**

**Patient name: _________________________________ Date: ___________________**

**1. _______________________________________________________________________**

**2. _______________________________________________________________________**

## Annex 6: Information and consent form for patients enrolled by CHWs.

**Name of Study:**

*Effectiveness of community level deployment of rapid diagnostic tests for malaria in Afghanistan: Cluster Randomised Trial.*

**Name of Investigators:**

London School of Hygiene and Tropical Medicine (LSHTM): Dr Mark Rowland, Dr Toby Leslie, Amy Mikhail

Health Protection and Research Organisation: Dr Ismail Mayan

HealthNet TPO: Dr M. Anwar Hasan Zai, Dr M Nader

Merlin: Dr Sayed Habib Bakhtash, Dr Sayed Hussein Hashimi.

**Research Information:**

1. The study aims to examine ways in which diagnosis and treatment for malaria and non-malaria fevers can be improved.
2. We are asking you to give consent to be included in this research study to examine the effectiveness of diagnosis and treatment for your disease.
3. If you agree to be in the study this is what will happen:
   1. I will ask you some questions and note your information on a form.
   2. I will ask you to provide me with a small blood sample which we will use to test your blood for malaria.
   3. Collecting the blood sample is done by using a lancet to prick the tip of your finger – this procedure will be conducted using a clean, single use lancet and I have been trained to perform the procedure. I will use clean gloves to reduce the risk of infection. The procedure will cause minor pain at the puncture site and there is a very small risk of secondary infection at the puncture site. I will use antiseptic to minimize this risk.
   4. Once I have collected the blood sample, and provided you with treatment your participation in the study is almost over.
   5. However, in case you go to the clinic because of this illness I will give you an identification card which you should show to the doctor or any other staff in the health centre. This will identify to them that you have participated in this study.
   6. If you do go to the health centre, the doctor will ask you to give consent to continue in the study and will explain what will happen in the clinic.
4. Your participation in the study is voluntary so you do not have to be enrolled. If you do not agree to be enrolled, I will treat you according to national treatment practices. If you do not want to participate this will not affect your treatment or access to healthcare now or in the future.
5. Any information that I collect from you will be treated confidentially.
6. The information recorded on the form will only note a number, and not your name. The information will be passed to the Merlin / HealthNet office in this province where it will be put into computer format. From there the information from all the patients will be sent to Kabul for analysis.
7. The blood sample that we collect will be sent to Kabul for analysis in our laboratory which will examine whether you blood has malaria or not. Because this takes some time, we will not be able to bring the result of the test back to you – it can take up to 4 weeks to process the samples.
8. If the laboratory in Kabul cannot conduct the analysis for any reason, we may send the samples to an overseas laboratory in London, UK.
9. The blood sample will be anonymous and may be stored for use in later research studies. The blood sample will only be marked with a number and so will not be traceable to you and your identity will not be known.
10. The blood sample will be stored at the HPRO office in Kabul, or at LSHTM in London. If you do not agree to the storage of your blood sample after this study is finished, we will destroy the sample after it is used.
11. Do you understand what I have said?
12. Do you have any questions?
13. Would you like to participate in the study?

If you have any queries on this form, contact:

Nangahar: Dr M Anwar (HealthNet TPO Project Coordinator): +93789880582

Kunduz: Dr Bakhtash (Merlin Project Coordinator): +93700712098

HPRO Project Manager: Dr Ismail Mayan: +93789435379

**CONSENT FORM:**

**Name of Study:**

*Effectiveness of community level deployment of rapid diagnostic tests for malaria in Afghanistan: Cluster Randomised Trial.*

**Name of Investigators:**

London School of Hygiene and Tropical Medicine (LSHTM): Dr Mark Rowland, Dr Toby Leslie, Amy Mikhail

Health Protection and Research Organisation: Dr Ismail Mayan

HealthNet TPO: Dr M. Anwar Hasan Zai, Dr M Nader

Merlin: Dr Sayed Habib Bakhtash, Dr Sayed Hussein Hashimi.

[pre-printed]

**Patient Number:**

**Day of enrolment:**

| **Sat** | **Sun** | **Mon** | **Tue** | **Wed** | **Thur** | **Fri 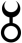** |
| --- | --- | --- | --- | --- | --- | --- |
|  |  |  |  |  |  |  |

**The study is approved by the ethics board of the Ministry of Public Health, Afghanistan and London School of Hygiene and Tropical Medicine.**

***CHW:***

I certify that I have explained the above to this patient and that she/he understood what I said and she/he agreed to participate in the study.

Signature …………………………… Date ………………………..

[Name]

***Patient or Patient’s Parent/Guardian:***

I have understood the verbal explanation given to me by _[CHW name]___________________ and I understand what my participation involves. I understand that my participation is voluntary and that I can withdraw my consent at any time.

I agree to take part in the study: Y [ ] N [ ]

I agree that my anonymous blood sample can be stored for use in other research studies at a later date:

Y [ ] N [ ]

Signature or Mark …………………… Date ………………………..

**Relationship to patient (if under 16 years): ..............................................**

If you have any queries on this form or the study, please contact:

Nangahar: Dr M Anwar (HealthNet TPO Project Coordinator): +93789880582

Kunduz: Dr Bakhtash (Merlin Project Coordinator): +93700712098

HPRO Project Manager: Dr Ismail Mayan: +93789435379

## Annex 7: Information and consent form for patients enrolled at clinics.

**Name of Study:**

*Effectiveness of community level deployment of rapid diagnostic tests for malaria in Afghanistan: Cluster Randomised Trial.*

**Name of Investigators:**

London School of Hygiene and Tropical Medicine (LSHTM): Dr Mark Rowland, Dr Toby Leslie, Amy Mikhail

Health Protection and Research Organisation: Dr Ismail Mayan

HealthNet TPO: Dr M. Anwar Hasan Zai, Dr M Nader

Merlin: Dr Sayed Habib Bakhtash, Dr Sayed Hussein Hashimi.

**Research Information:**

1. The study aims to examine ways in which diagnosis and treatment for malaria and non-malaria fevers can be improved.
2. We are asking you to give consent to be included in this research study to examine the effectiveness of diagnosis and treatment for your disease.
3. If you agree to be in the study this is what will happen:
   1. I will ask you some questions about you and your condition and note your information on a form. I will also note the treatment that is given
   2. I will ask you to provide me with a small blood sample which we will use to test your blood for malaria.
   3. Collecting the blood sample is done by using a lancet to prick the tip of your finger – this procedure will be conducted using a clean, single use lancet and I have been trained to perform the procedure. I will use clean gloves to reduce the risk of infection. The procedure will cause minor pain at the puncture site and there is a very small risk of secondary infection at the puncture site. I will use antiseptic to minimize this risk.
   4. Once I have collected the blood sample, and provided you with treatment your participation in the study is almost over.
4. Your participation in the study is voluntary so you do not have to take part. If you do not agree to participate, I will treat you according to national treatment practices. If you do not want to participate this will not affect your treatment or access to healthcare now or in the future.
5. Any information that I collect from you will be treated confidentially.
6. The information recorded on the form will only note a number, and not your name. The information will be passed to the Merlin / HealthNet office in this province where it will be put into computer format. From there the information from all the patients will be sent to Kabul for analysis.
7. If you were previously enrolled in the study as a patient, we will link this information to your previous number in that study.
8. The blood sample that we collect will be sent to Kabul for analysis in our laboratory which will examine whether you blood has malaria or not. However, we will not normally be able to send the result of the test back to you – it can take up to 4 weeks to process the samples, and the sample will not have your name on it.
9. If the laboratory in Kabul cannot conduct the analysis for any reason, we may send the samples to an overseas laboratory.
10. The blood sample will be anonymous and may be stored for use in later research studies. The blood sample will only be marked with a number and so will not be traceable to you and your identity will not be known.
11. The blood sample will be stored at the HPRO office in Kabul, or at LSHTM in London. If you do not agree to the storage and use of your blood sample after this study is finished, we will destroy the sample after it is used.
12. Do you understand what I have said?
13. Do you have any questions?
14. Would you like to participate in the study?

If you have any queries on this form or the study, please contact:

Nangahar: Dr M Anwar (HealthNet TPO Project Coordinator): +93789880582

Kunduz: Dr Bakhtash (Merlin Project Coordinator): +93700712098

HPRO Project Manager: Dr Ismail Mayan: +93789435379**CONSENT FORM:**

**Name of Study:**

*Effectiveness of community level deployment of rapid diagnostic tests for malaria in Afghanistan: Cluster Randomised Trial.*

**Name of Investigators:**

London School of Hygiene and Tropical Medicine (LSHTM): Dr Mark Rowland, Dr Toby Leslie, Amy Mikhail

Health Protection and Research Organisation: Dr Ismail Mayan

HealthNet TPO: Dr M. Anwar Hasan Zai, Dr M Nader

Merlin: Dr Sayed Habib Bakhtash, Dr Sayed Hussein Hashimi.

**Patient Number:**

[pre-printed]

**The study is approved by the ethics board of the Ministry of Public Health, Afghanistan and London School of Hygiene and Tropical Medicine.**

***Study Registrar / Clinician:***

I certify that I have explained the above to this patient and that she/he understood what I said and she/he agreed to participate in the study.

Signature …………………………… Date ………………………..

[Name]

***Patient or Patient’s Parent/Guardian:***

I have understood the verbal explanation given to me by ____________________ and I understand what my participation involves. I understand that my participation is voluntary and that I can withdraw my consent at any time.

I agree to take part in the study: Y [ ] N [ ]

I agree that my blood sample can be stored anonymously for use in other research studies at a later date:

Y [ ] N [ ]

Signature or Mark …………………… Date ………………………..

**Relationship to patient (if under 16 years): ..............................................**

**Was the patient enrolled into the CRT by CHW?** N [ ] Y [ ] – if yes, note the ID number:

If you have any queries on this form or the study, please contact:

Nangahar: Dr M Anwar (HealthNet TPO Project Coordinator): +93789880582

Kunduz: Dr Bakhtash (Merlin Project Coordinator): +93700712098

HPRO Project Manager: Dr Ismail Mayan: +93789435379

## Annex 8: Consent form for CHW Participation:

**Name of Study:**

*Effectiveness of community level deployment of rapid diagnostic tests for malaria in Afghanistan: Cluster Randomised Trial.*

**Name of Investigators:**

London School of Hygiene and Tropical Medicine (LSHTM): Dr Mark Rowland, Dr Toby Leslie, Amy Mikhail

Health Protection and Research Organisation: Dr Ismail Mayan

HealthNet TPO: Dr M. Anwar Hasan Zai, Dr M Nader

Merlin: Dr Sayed Habib Bakhtash, Dr Sayed Hussein Hashimi.

**CHW Name:**

**CHW Clinic Name:**

**Information:**

1. You have already attended the training which described your participation in the study.
2. By signing below, you agree that you will collect information about patients that you see during your duties as community health workers.
3. You understand that the recorded information will be used to examine the decisions that you make during the consultation with patients.
4. You understand that the information that we collect is confidential and we will never disclose your identity to anyone outside the research staff.

**CONSENT FORM:**

***Trainer / CHS:***

I certify that I have explained the above to the CHW and that she/he understood what I said and she/he agreed to participate in the study.

Signature …………………………… Date ………………………..

[Name]

***CHW:***

I have understood the training and explanation given to me by ____________________ and I understand what my participation involves. I understand that my participation is voluntary and that I can withdraw my consent at any time.

I agree to take part in the study: Y [ ] N [ ]

Signature or Mark …………………… Date ………………………..

If you have any queries on this form or the study, please contact:

Nangahar: Dr M Anwar (HealthNet TPO Project Coordinator): +93789880582

Kunduz: Dr Bakhtash (Merlin Project Coordinator): +93700712098

HPRO Project Manager: Dr Ismail Mayan: +93789435379

1. Kohistani , Nadeeb, Leslie. Home [sic – community] based management of malaria through the BDN program in North-East and Eastern regions of Afghanistan. TDR Small Grants Scheme Technical Report, 2008, unpublished report. [↑](#footnote-ref-1)
2. Conducted by WHO through funding from USAID in the Northern Region. [↑](#footnote-ref-2)
3. The restriction factor was defined as the proportion, $p=1-\frac{n}{N}$, where n is the total number of acceptable allocations from N randomly generated allocations (N=10,000) [↑](#footnote-ref-3)
4. Integrated management of childhood illness: caring for newborns and children in the

   community. WHO, 2010 [↑](#footnote-ref-4)
